# GOLM1 restricts colitis and colon tumorigenesis by ensuring Notch signaling equilibrium in intestinal homeostasis

**DOI:** 10.1038/s41392-021-00535-1

**Published:** 2021-04-14

**Authors:** Yang Pu, Ya Song, Mengdi Zhang, Caifeng Long, Jie Li, Yanan Wang, Yinzhe Xu, Fei Pan, Na Zhao, Xinyu Zhang, Yanan Xu, Jianxin Cui, Hongying Wang, Yan Li, Yong Zhao, Di Jin, Hongbing Zhang

**Affiliations:** 1 State Key Laboratory of Medical Molecular Biology, Department of Physiology, Institute of Basic Medical Sciences and School of Basic Medicine, Peking Union Medical College and Chinese Academy of Medical Sciences, Beijing, China; 2 Institute of Cancer Stem Cell, Dalian Medical University, Dalian, Liaoning China; 3 Chinese PLA General Hospital, Beijing, China; 4 State Key Laboratory of Biomembrane and Membrane Biotechnology, Institute of Zoology, Chinese Academy of Sciences, Beijing, China; 5 State Key Laboratory of Molecular Oncology, National Cancer Center/National Clinical Research Center for Cancer/Cancer Hospital, Chinese Academy of Medical Sciences and Peking Union Medical College, Beijing, China; 6 Department of Anatomy and Physiology, College of Basic Medical Sciences, Shanghai Jiao Tong University, Shanghai, China

**Keywords:** Gastrointestinal cancer, Cancer

## Abstract

Intestinal epithelium serves as the first barrier against the infections and injuries that mediate colonic inflammation. Colorectal cancer is often accompanied with chronic inflammation. Differed from its well-known oncogenic role in many malignancies, we present here that Golgi membrane protein 1 (GOLM1, also referred to as GP73) suppresses colorectal tumorigenesis via maintenance of intestinal epithelial barrier. GOLM1 deficiency in mice conferred susceptibility to mucosal inflammation and colitis-induced epithelial damage, which consequently promoted colon cancer. Mechanistically, depletion of GOLM1 in intestinal epithelial cells (IECs) led to aberrant Notch activation that interfered with IEC differentiation, maturation, and lineage commitment in mice. Pharmacological inhibition of Notch pathway alleviated epithelial lesions and restrained pro-tumorigenic inflammation in GOLM1-deficient mice. Therefore, GOLM1 maintains IEC homeostasis and protects against colitis and colon tumorigenesis by modulating the equilibrium of Notch signaling pathway.

## Introduction

Colorectal cancer (CRC) is the third leading cause of cancer-related death, with ~1.8 million new cases diagnosed worldwide in 2018.^1^ Patients with inflammatory bowel diseases (IBD), such as Crohn’s disease or ulcerative colitis (UC), are more susceptible to CRC. This type of CRC is referred as colitis-associated colorectal cancer (CAC),^2^ which causes 10–15% of the annual deaths in IBD patients.^3–5^ Among the various causes of colitis and CAC, the disrupted intestinal barrier is an important predisposing factor for disease progression.^6^ The functional coordination of the intestinal barrier depends on the integrity of the epithelial layer and host immune responses. A compromised intestinal barrier leads to the host susceptibility of intestinal injury.^7^ The repetitive injuries result in sustained inflammation, excessive tissue regeneration, and hyperplasia which eventually promote colonic carcinogenesis.^8,9^ Indeed, mice deficient in epithelial barrier have shown increased vulnerability to colitis and CAC.^10–13^ Despite the known importance of the intestinal epithelial barrier in colorectal tumorigenesis, its underlying molecular mechanisms in tumor formation and development remain unclear.

GOLM1 (Golgi membrane protein 1, also referred to as GP73 or Golph2), a Golgi type II transmembrane protein, expresses variedly in different tissues.^14^ Overexpressed GOLM1 has been recognized as an oncogenic protein in many malignancies, including hepatocellular carcinoma,^15^ prostate cancer,^16^ lung cancer,^17–19^ breast cancer,^20^ renal cancer^21^, and glioblastoma.^16^ Despite these findings, its impact on CRC has yet to be elucidated.

GOLM1 has been implicated in inflammation and immunoregulation. Genetic inactivation of GOLM1 in myeloid cells suppresses interleukin (IL)-12 secretion and polarizes marcophage towards M2 type.^22^ Moreover, GOLM1 overexpression has been identified in virus-associated inflammatory liver diseases.^23^ GOLM1 also promotes HCV replication by inhibiting type I interferon production.^22^ The potential role of GOLM1 in colonic inflammatory diseases is, however, unclear.

In this study, we first analyzed the expression of GOLM1 in patients’ tissues to investigate whether GOLM1 was potentially implicated in the pathogenesis of colitis and colon cancer. We then generated systemic and epithelial-specific *Golm1* knockout mice as well as bone marrow chimera transplantation models to determine the involvement of GOLM1 in colitis and CAC. We further revealed the physiological role of GOLM1 in maintaining the intestinal epithelial barrier and preventing pro-tumorigenic inflammation in colons. Lastly, we alleviated GOLM1 depletion-mediated colonic disorder by targeting the GOLM1-Notch signaling cascades we dissected.

## Results

### GOLM1-deficient mice are predisposed to AOM/DSS-induced colon tumorigenesis

**Fig. 1:**
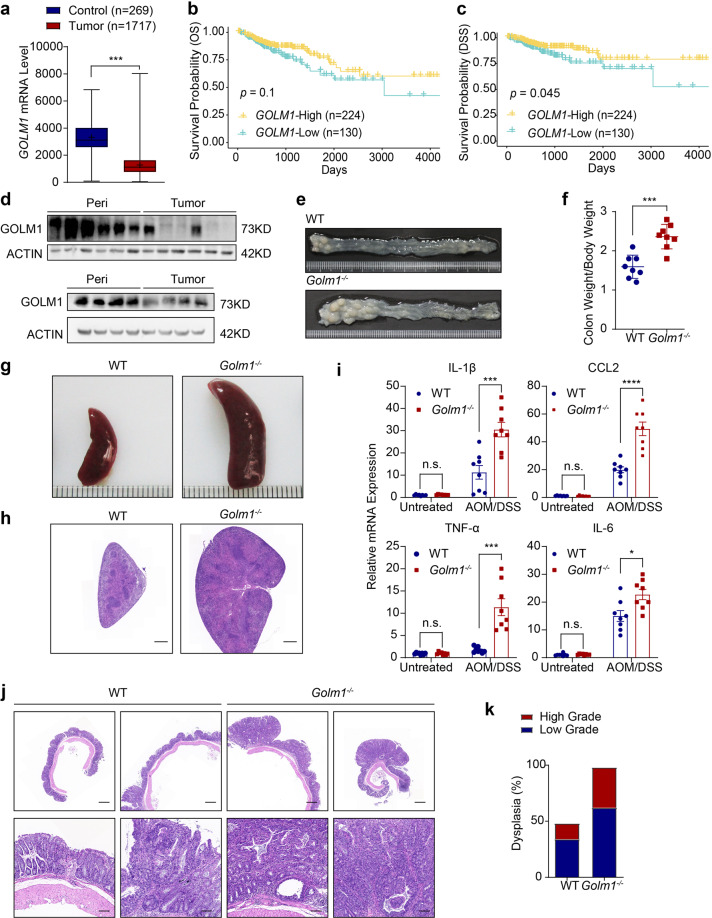
GOLM1-deficient mice are more susceptible to AOM/DSS-induced colon tumorigenesis. **a** *GOLM1* expression in human colorectal cancer (CRC) tissues compared with that in normal tissues using expression data from the TCGA database (***P < 0.001; unpaired, two-tailed Student’s *t* test). **b** Curves for overall survival are shown between high and low expression of *GOLM1* in CRC samples based on the TCGA database. **c** Curves for disease-specific survival are shown between high and low expression of *GOLM1* in CRC samples based on the TCGA database. **d** Human CRC and adjacent normal tissues we collected were extracted and immunoblotted (*n* = 10). **e** Representative images of colon tumors obtained from AOM/DSS-treated mice. **f** The average ratio of colon weight/body weight in AOM/DSS-treated mice (each symbol in each column represents an individual mouse, *n* = 8; the data are represented as the means ± SEM; ***P < 0.01; unpaired, two-tailed Student’s *t* test). **g** Representative images of the spleen obtained from AOM/DSS-treated mice. **h** Representative Hematoxylin and Eosin (H&E) staining of mouse spleen sections obtained from AOM/DSS-treated mice. Scale bars, 1000 μm. **i** Relative mRNA expression levels of inflammatory mediators in the distal colon of AOM/DSS-treated mice determined by quantitative reverse transcription PCR (qRT-PCR). Relative expression reflects the fold change calculated by comparing with the average expression levels in untreated WT mice (the data are represented as the means ± SEM, *n* = 8; ***P < 0.001, *P < 0.05; unpaired, two-tailed Student’s *t* test). **j** Representative H&E staining of mouse colon sections obtained from AOM/DSS-treated mice. Upper scale bars, 500 μm; lower scale bars, 50 μm. **k** Percentages of mice with dysplasia at 70 days after AOM injection administration

By comparing *GOLM1* mRNA expression in normal and cancerous colon tissues from human patient biopsies based on TCGA database, we found *GOLM1* expression was significantly lower in the tumor tissues than in normal colonic tissues (Fig. 1a). Although the correlation of low *GOLM1* expression and poor overall survival did not reach statistical significance, disease-specific survival analysis suggested that low *GOLM1* expression was significantly associated with poor prognosis of CRC patients of TCGA database (Fig. 1b, c). Furthermore, we confirmed the decreased GOLM1 protein levels in clinical samples of CRC compared with their paired normal biopsies by immunoblotting (Fig. 1d).

To investigate the role of GOLM1 in colon cancer, we established AOM/DSS-induced CAC model in systemic *Golm1* knockout (*Golm1*^*−/−*^*)* mice and control mice (WT) (Supplementary Fig. 1a). While untreated *Golm1*^*−/−*^ mice exhibited no morphological abnormality (Supplementary Fig. 1b–d), the treated *Golm1*^*−/−*^ mice displayed an increased colon tumor burden and an enhanced colon to body weight ratio compared to WT mice (Fig. 1e, f and Supplementary Fig. 1e, f). Moreover, treated *Golm1*^*−/−*^ mice showed a greater extent of splenomegaly which was indicative of increased systemic inflammation (Fig. 1g, h). Consistently, the colonic mRNA levels of several proinflammatory cytokines, such as IL-1β, IL-6, CCL2, and TNF-α, were significantly higher in AOM/DSS-treated *Golm1*^*−/−*^ mice than in their WT counterparts (Fig. 1i). Histological analysis of the tumor-bearing colons revealed more low-grade and high-grade dysplasia in AOM/DSS-treated *Golm1*^*−/−*^ mice (Fig. 1j, k), suggesting that GOLM1 deficiency increased mouse susceptibility to CAC.

### GOLM1-deficient mice are susceptible to DSS-induced colitis

**Fig. 2:**
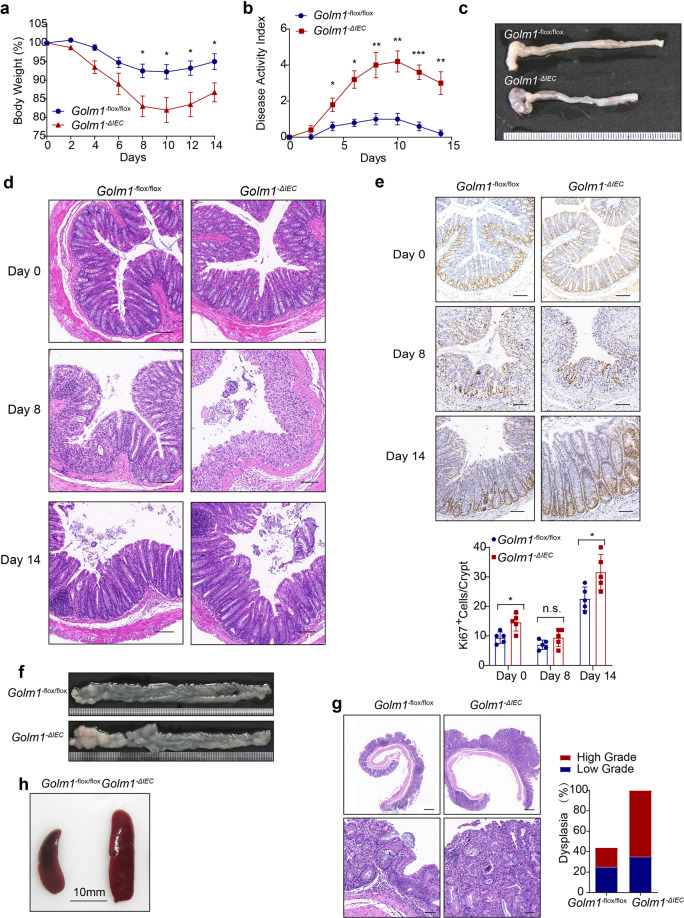
GOLM1 depletion renders mice vulnerable to DSS-induced colitis. **a** *GOLM1* expression was analyzed from intestinal mucosal biopsies of ulcerative colitis (UC) patients and controls of cohorts GSE87466 and GSE75214 (***P < 0.001, ****P < 0.0001; unpaired, two-tailed Student’s *t* test). **b** UC patients’ samples and adjacent normal tissues we collected were extracted and immunoblotted (*n* = 10). **c** Colon lysates from DSS-treated WT mice sacrificed on the indicated days were analyzed by immunoblotting with indicated antibodies (*n* = 5). **d** Mice were administered with 2% DSS for 5 days. The mouse body weights were recorded on indicated days (the data are represented as the means ± SEM, *n* = 5; *P < 0.05, **P < 0.01). **e** Representative images of colons from mice treated with 2% DSS for 5 days and sacrificed on day 8. **f** The disease activity indexes of mice administered 2% DSS treatment for 5 days (the data are represented as the means ± SEM, *n* = 5; *P < 0.05, **P < 0.01). **g** Representative H&E staining of mouse colon sections obtained from DSS-treated mice on the indicated days. Scale bars, 100 μm. The sections were histologically graded for epithelial damage (the data are represented as the means ± SEM; *P < 0.05, **P < 0.01; unpaired, two-tailed Student’s *t* test). **h** Representative CD45, F4/80, and CD3 staining of the colon sections obtained from mice treated with 2% DSS and sacrificed on day 8. Scale bars, 100 μm. **i** The relative mRNA expression levels of inflammatory mediators in the distal colon of DSS-treated mice were determined by qRT-PCR (the data are represented as the means ± SEM, *n* = 5; *P < 0.05, **P < 0.01, ***P < 0.001; unpaired, two-tailed Student’s *t* test). The relative expression reflects the fold change, which was calculated by comparing with the average expression levels in untreated WT mice. **j** Colon lysates from DSS-treated mice sacrificed on day 8 were analyzed by immunoblotting (*n* = 5) with the indicated antibodies

Since AOM-induced phosphorylation levels of H2AX and p53 were similar in AOM-treated WT and *Golm1*^*−/−*^ mice colons (Supplementary Fig. 2a), the differences in tumorigenesis between WT and *Golm1*^*−/−*^ mice following AOM/DSS administration did not stem from the initial DNA damage responses. To investigate the role of GOLM1 in colonic inflammation, we analyzed *GOLM1* expression in intestinal biopsies from UC patients of Li’s cohort (GSE 87466) and Arijs’s cohort (GSE75214). *GOLM1* expression was significantly decreased in UC patients (Fig. 2a). Moreover, lower expression of GOLM1 was further confirmed by immunoblotting in the UC patients’ samples we collected (Fig. 2b). Consistently, colonic GOLM1 expression was considerably decreased in DSS-treated WT mice than in vehicle-treated counterparts (Fig. 2c and Supplementary Fig. 2b). All *Golm1*^*−/−*^ mice treated with 3% DSS succumbed to colitis 14 days after DSS induction while >80% of their WT counterparts survived (Supplementary Fig. 2c). To keep all the mice alive for further detailed analysis, we decreased DSS concentration to 2% in subsequent experiments. Compared to WT mice, *Golm1*^*−/−*^ mice lost more body weight and exhibited a higher disease activity index (DAI) with shorter colons after DSS treatment (Fig. 2d–f). Furthermore, DSS-treated *Golm1*^*−/−*^ mice exhibited much more severe colonic epithelial damage and inflammatory infiltration with almost complete crypt loss and erosion than WT counterparts (Fig. 2g, h). Accordingly, the intestinal expressions of inflammatory cytokines and chemokines, such as IL-6, IL-1β, TNF-α, and CCL2 were remarkably upregulated in *Golm1*^*−/−*^ mice compared with WT mice following DSS administration (Fig. 2i). Moreover, DSS-treated *Golm1*^*−/−*^ mice showed elevated p65 and STAT3 phosphorylation (Fig. 2j), which are indicative of robust activation of the NF-κB and IL-6/STAT3 signaling pathways, respectively.^24^ Taken together, the enhanced inflammatory responses in *Golm1*^*−/−*^ mice with DSS-induced colonic epithelial damage may contribute to colitis and its progression to CAC.

### GOLM1 deficiency in IECs increases mouse susceptibility to DSS-induced colitis and AOM/DSS-induced CAC

To understand the mechanisms involved in the colitis and CAC vulnerability of *Golm1*^*−/−*^ mice, we differentiated the contributions of hematopoietic cells from those of non-hematopoietic cells in colitis progression in *Golm1*^*−/−*^mice with bone marrow chimera transplantation (Supplementary Fig. 3a). WT mice that received transfers of WT or *Golm1*^*−/−*^ mouse bone marrow cells (WT/WT or *Golm1*^*−/−*^/WT) displayed similar sensitivities to DSS in terms of body weight loss and DAI score. However, *Golm1*^*−/−*^ recipients (WT/*Golm1*^*−/−*^or *Golm1*^*−/−*^/*Golm1*^*−/−*^) exhibited more severe disease progression and epithelial damage than WT recipients after DSS administration (Supplementary Fig. 3b–d). Colitis-associated pathology observed in *Golm1*^*−/−*^ mice is thus mainly due to GOLM1 deficiency in non-hematopoietic cells.

**Fig. 3:**
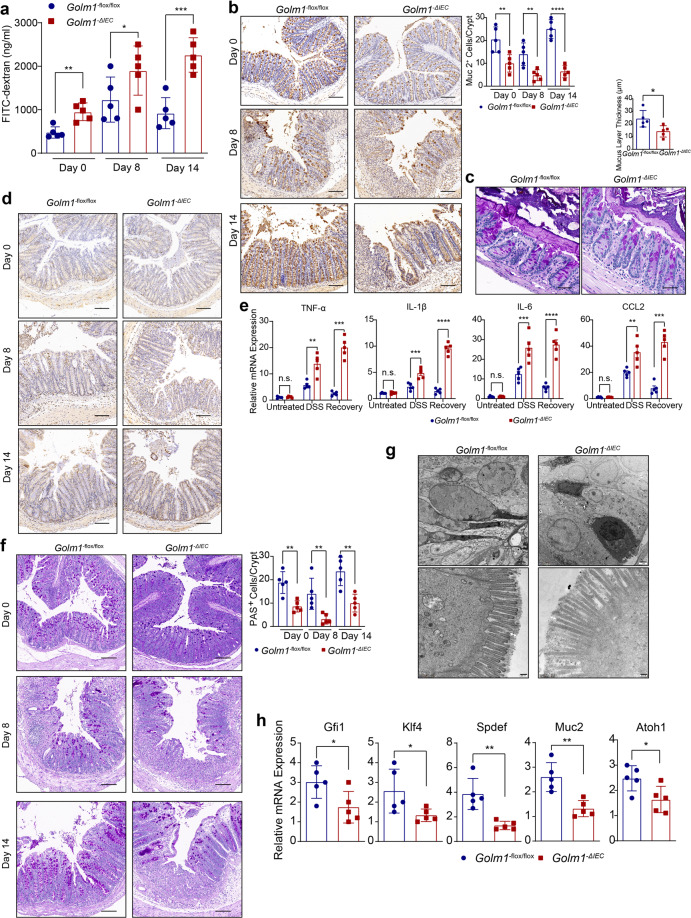
GOLM1 deficiency in IEC increases mouse susceptibility of colitis and CAC. **a** The body weights of 2% DSS-treated *Golm1*^*−ΔIEC*^ mice and *Golm1*^*−flox/flox*^ counterparts were recorded on indicated days (the data are represented as the means ± SEM, *n* = 5; *P < 0.05). **b** The disease activity indexes of *Golm1*^*−ΔIEC*^ mice and *Golm1*^*−flox/flox*^ counterparts administered 2% DSS for 5 days (the data are represented as the means ± SEM, *n* = 5; *P < 0.05, **P < 0.01, ***P < 0.001). **c** Representative image of colons obtained from *Golm1*^*−ΔIEC*^ mice and *Golm1*^*−flox/flox*^ counterparts treated with 2% DSS for 5 days and sacrificed on day 8. **d** Representative H&E staining of mouse colon sections obtained from DSS-treated mice on the indicated days. Scale bars, 100 μm. **e** Representative Ki67 staining of colon sections from mice treated with 2% DSS and sacrificed on the indicated days. Scale bars, 100 μm. Quantification is shown in the histogram (the data are represented as the means ± SEM, *n* = 5; *P < 0.05; unpaired, two-tailed Student’s *t* test). **f** Representative images of colon tumors obtained from AOM/DSS-treated mice. **g** Representative H&E staining of mouse colon sections obtained from AOM/DSS-treated mice. Upper scale bars, 500 μm; lower scale bars, 50 μm. Percentages of mice with dysplasia at 70 days after injection of AOM. **h** Representative image of the spleens obtained from AOM/DSS-treated mice

GOLM1 has been known to express in various non-hematopoietic cells, especially in epithelial cells, which are closely related to colitis progression.^25,26^ We speculated that robust basal GOLM1 expression in IECs might regulate intestinal inflammation. To this end, we generated mice expressing *Villin-Cre* and *Golm1-lox* alleles, termed *Golm1*^*−ΔIEC*^ mice, to specifically delete GOLM1 in IECs and verified the GOLM1 depletion efficacy by immunoblotting (Supplementary Fig. 3e, f). While there is no intestinal morphological difference between *Golm1*^*−ΔIEC*^ and *Golm1*^*−flox/flox*^ mice in steady-state, DSS treatment rendered *Golm1*^*−ΔIEC*^ mice more body weight loss and higher DAI scores (Fig. 3a, b and Supplementary Fig. 3g). The shortened colon and destructed intestinal epithelium further confirmed robust colitis progression in DSS-treated *Golm1*^−*ΔIEC*^ mice (Fig. 3c, d). Additionally, DSS caused more intestinal epithelial apoptosis in DSS-treated *Golm1*^*−ΔIEC*^ mice than in *Golm1*^*−flox/flox*^ counterparts, while untreated *Golm1*^−*ΔIEC*^ mice and *Golm1*^*−flox/flox*^ counterparts displayed similar levels of IECs apoptosis (Supplementary Fig. 3h). We then analyzed the BCL-2 family members, well-characterized regulators of apoptosis, in DSS-treated *Golm1*^−*ΔIEC*^ and *Golm1*^*−flox/flox*^ mice. The level of proapoptotic protein BAK in IECs was significantly elevated in DSS-treated *Golm1*^−*ΔIEC*^ mice, whereas the antiapoptotic proteins BCL2, BCl-XL, and MCL1 were expressed at similar levels in DSS-treated *Golm1*^*−ΔIEC*^ and *Golm1*^*−flox/flox*^ mice (Supplementary Fig. 3i). During the recovery phase after DSS administration, *Golm1*^*−ΔIEC*^ mice exhibited impaired intestinal recovery with more colonic proliferative cells and hyperplastic elongated crypts; while the *Golm1*^*−flox/flox*^ counterparts’ colons recovered almost completely (Fig. 3d, e). The uncontrolled IEC hyperproliferation in recovering DSS-treated *Golm1*^−*ΔIEC*^ mice is a compensatory response to augmented apoptosis, suggesting the susceptibility of *Golm1*^−*ΔIEC*^ mice to CAC.

As expected, *Golm1*^*−ΔIEC*^ mice developed more intestinal tumors and exhibited enhanced tumor burden compared with *Golm1*^*−flox/flox*^ mice when treated with AOM/DSS (Fig. 3f, g and Supplementary Fig. 3j, k). Similar to AOM/DSS-treated *Golm1*^*−/−*^ mice, the increased splenomegaly and colonic inflammatory mediators in AOM/DSS-treated *Golm1*^*−ΔIEC*^ mice indicated enhanced systemic inflammation (Fig. 3h and Supplementary Fig. 3l). These results suggest that loss of GOLM1 in IEC contributes to robust colitis and increased CAC.

### Goblet cell loss and intestinal barrier dysfunction contribute to GOLM1 deficiency-mediated exacerbated colitis and CAC

**Fig. 4:**
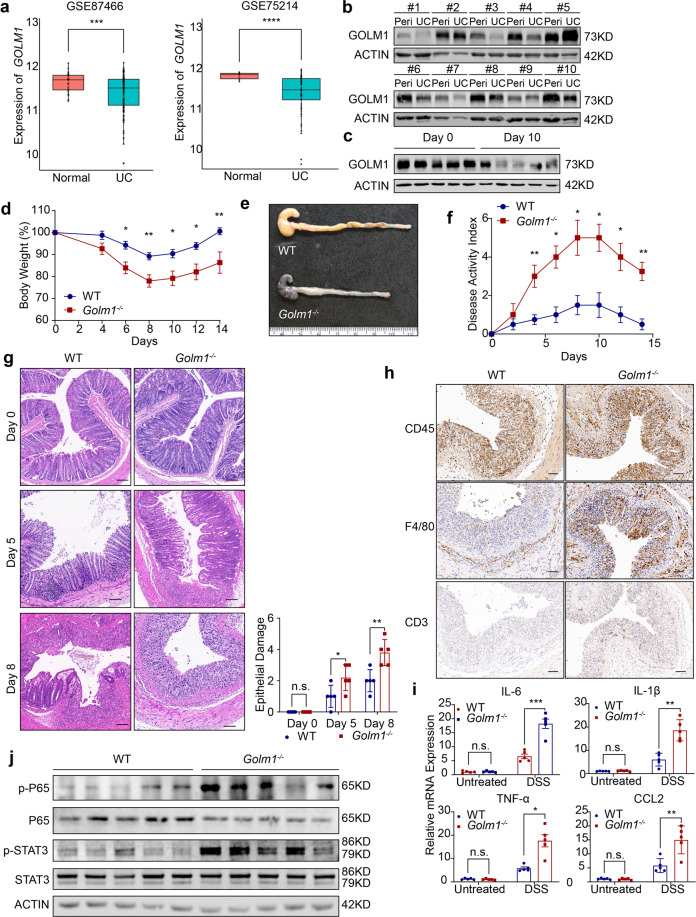
GOLM1 restricts colitis and CAC through maintaining goblet cells and intestinal barrier. **a** Intestinal permeability was measured by concentration of FITC-dextran in mouse serum (the data are represented as the means ± SEM, *n* = 5; *P < 0.05, **P < 0.01, ***P < 0.0001; unpaired, two-tailed Student’s *t* test). **b** Representative MUC-2 staining of mouse colon sections obtained from mice treated with 2% DSS and sacrificed on indicated days. Scale bars, 100 μm. Quantification is shown in the histogram (the data are represented as the means ± SEM, *n* = 5; **P < 0.01, ****P < 0.0001; unpaired, two-tailed Student’s *t* test). **c** Representative PAS staining of mouse colon sections obtained from *Golm1*^*−ΔIEC*^ \and *Golm1*^*−flox/flox*^ mice. White arrows indicate innate mucus layer. Scale bars, 20 μm. Quantification is shown in the histogram (the data are represented as the means ± SEM, *n* = 5; *P < 0.05; unpaired, two-tailed Student’s *t* test). **d** Representative CD3 staining of mouse colon sections obtained from mice treated with 2% DSS and sacrificed on indicated days. Scale bars, 100 μm. **e** Relative mRNA expression levels of inflammatory mediators in the distal colon of DSS-treated mice determined by qRT-PCR. Relative expression reflects the fold change calculated by comparing with the average expression levels in untreated *Golm1*^*−flox/flox*^ mice (the data are represented as the means ± SEM, *n* = 5; *P* < 0.05, **P < 0.01, ***P < 0.001, ****P < 0.0001; unpaired, two-tailed Student’s *t* test). **f** Representative PAS staining of mouse colon sections obtained from DSS-treated mice. Scale bars, 100 μm. Quantification is shown in the histogram (the data are represented as the means ± SEM, *n* = 5; **P < 0.01; unpaired, two-tailed Student’s *t* test). **g** Representative electron microscopy images of goblet cells and epithelial glycocalyx produced by goblet cells between the intestinal microvilli from the colons of *Golm1*^*−ΔIEC*^ and *Golm1*^*−flox/flox*^ mice. Scale bars, 100 nm (upper panel), 200 nm (lower panel). White arrows indicate mucopolysaccharide matrix (epithelial glycocalyx). **h** Relative mRNA expression levels of the indicated genes in IECs isolated from *Golm1*^*−ΔIEC*^ and *Golm1*^*−flox/flox*^ mice determined by qRT-PCR (the data are represented as the means ± SEM, *n* = 5; *P < 0.05, **P < 0.01; unpaired, two-tailed Student’s *t* test)

The intestinal epithelial barrier serves as the prime protective layer from luminal antigens and their impairment has been frequently linked with IBD and CAC.^5^ We detected increased intestinal permeability of *Golm1*^*−ΔIEC*^ mice not only after DSS administration but also in the steady-state, which indicated that the intestinal barrier impairment already existed in untreated *Golm1*^*−ΔIEC*^ mice (Fig. 4a). Pre-treatment using a well-studied probiotic mixture, VSL#3, decreased the intestinal permeability and alleviated the body weight loss and epithelial damage in DSS-treated *Golm1*^*−ΔIEC*^ mice (Supplementary Fig. 4a–c). This finding further confirmed that intestinal barrier defects have functionally causal relationship with the colitis progression in DSS-treated *Golm1*^*−ΔIEC*^ mice. Consistent with the reduced mucus layer thickness, MUC-2, the most abundant component of the intestinal barrier, was also decreased in *Golm1*^*−ΔIEC*^ mouse colons in steady state (Fig. 4b, c). Moreover, we observed decreased MUC-2 expression in both DSS-treated *Golm1*^*−ΔIEC*^ and *Golm1*^*−flox/flox*^ mice compared with their respective control mice (Fig. 4b). MUC-2 expression then recovered to basal levels in recovering *Golm1*^*−flox/flox*^ mice but not in the recovering *Golm1*^*−ΔIEC*^ mice. Increased and sustained immune cells infiltration and inflammatory cytokines expressions were observed in DSS-treated *Golm1*^*−ΔIEC*^ mice (Fig. 4d, e). The recovery inability of mucus production and barrier integrity may underlie the sustained inflammation in *Golm1*^*−ΔIEC*^ mice after DSS administration. The pro-tumorigenic microenvironment together with the ensuing IECs hyperproliferation contribute to the hyperplasia and CAC initiation. We observed that rebamipide, a mucosal repairing agent, could reduce AOM/DSS-induced tumorigenesis in *Golm1*^*−*^^*Δ*^^*IEC*^ mice (Supplementary Fig. 4d–h). Thus, MUC-2 loss-mediated mucosal barrier impairment is a causal factor rather than a consequence of colitis and CAC vulnerability in *Golm1*^*−ΔIEC*^ mice.

Goblet cells are responsible for MUC-2 production. Colons from *Golm1*^*−ΔIEC*^ mice presented significantly fewer goblet cells than those from *Golm1*^*−flox/flox*^ mice (Fig. 4f). Electron microscopy showed less epithelial glycocalyx and abnormal nucleus morphology with absent secretory vacuoles in goblet cells of *Golm1*^*−ΔIEC*^ mouse colons but dense squeezed nucleus and enriched secretory vacuoles with mucins in goblet cells of *Golm1*^*−flox/flox*^ mouse colons (Fig. 4g). Analysis of IEC mRNA expression levels confirmed the downregulation of goblet cell differentiation/maturation governing transcript markers (*Atoh1, Gfi1, Spdef, Klf4, Muc2)* in *Golm1*^*−ΔIEC*^ mice (Fig. 4h). These observations suggest that GOLM1 deficiency in IECs results in decreased goblet cells and defective goblet cell maturation.

Although epithelial tight junction (TJ) stability is considered as another crucial factor of modulating intestinal permeability and epithelial integrity, we found no difference in the expression of key genes involved in TJ regulation and no significant morphological changes of TJ between *Golm1*^*−ΔIEC*^ and *Golm1*^*−flox/flox*^ mice (Supplementary Fig. 4i, j). Taken together, the intestinal epithelial barrier defects in *Golm1*^*−ΔIEC*^ mice mainly result from the loss of matured goblet cells which are essential for mucin secretion and colonic mucus layer integrity.

### Loss of GOLM1 in IECs alters IEC lineage specification and differentiation via upregulating Notch signaling

**Fig. 5:**
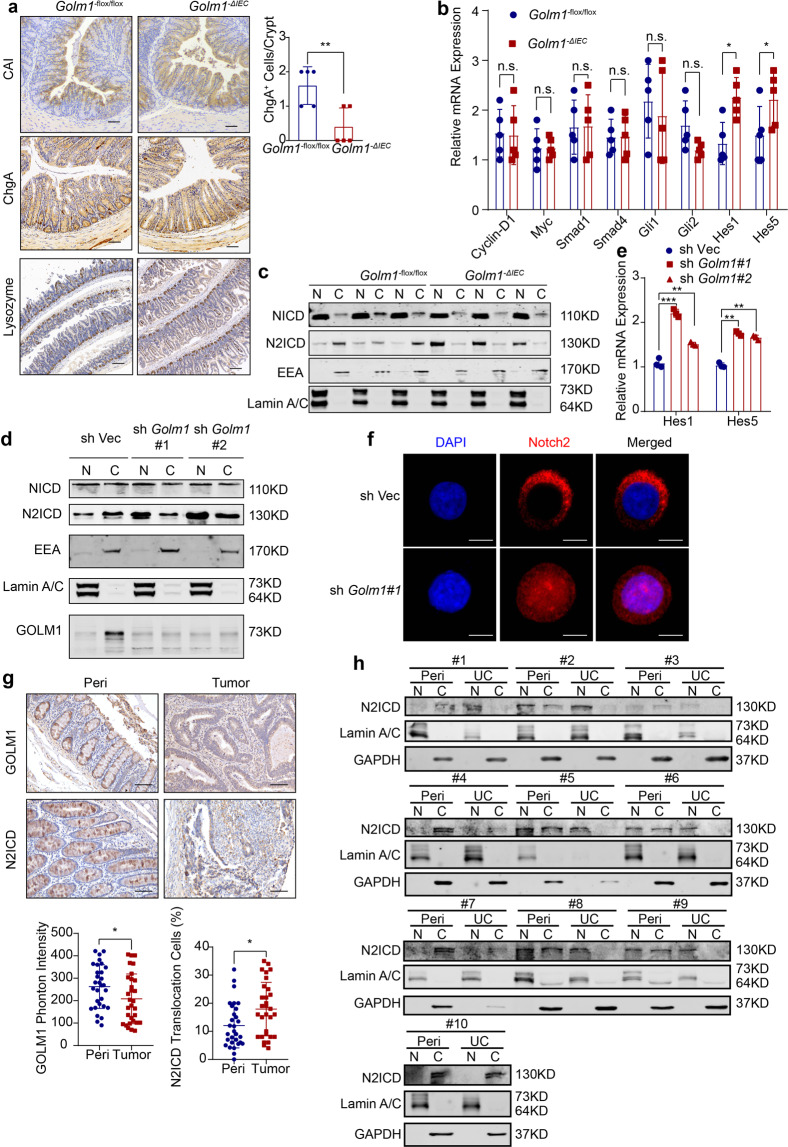
GOLM1 deletion causes the nuclear translocation of N2ICD. **a** Representative CAI and ChgA staining of mouse colon sections and representative Lysozyme staining of mouse small intestine sections obtained from untreated *Golm1*^*−ΔIEC*^ and *Golm1*^*−flox/flox*^ mice. Scale bars, 100μm. Quantification of ChgA staining is shown in the histogram (the data are represented as the means ± SEM, *n* = 5; *P < 0.05; unpaired, two-tailed Student’s *t* test). **b** The relative mRNA levels of genes that represent the major differentiated signaling pathways in isolated IECs from untreated *Golm1*^*−ΔIEC*^ and *Golm1*^*−flox/flox*^ mice were determined by qRT-PCR (the data are represented as the means ± SEM, *n* = 5; *P < 0.05; unpaired, two-tailed Student’s *t* test). **c** The separated cytoplasmic and nuclear lysates of IECs isolated from untreated *Golm1*^*−ΔIEC*^ mice and *Golm1*^*−flox/flox*^ counterparts were analyzed by immunoblotting (*n* = 3) with indicated antibodies. **d** Cellular fractionations from GOLM1-deficient Caco-2 cells and control cells were analyzed by immunoblotting with indicated antibodies. **e** The relative mRNA expression levels of Notch downstream genes in GOLM1-deficient Caco-2 cells and control cells were determined (the data are represented as the means ± SEM, ***P < 0.001; unpaired, two-tailed Student’s *t* test). **f** Representative immunofluorescence staining for N2ICD in GOLM1-deficient Caco-2 cells and control cells. Scale bars, 20 μm. **g** Representative immunochemistry staining for GOLM1 and N2ICD in 30 CRC samples of tissue microarray. Scale bars, 50 μm. IHC images were calculated using Image-Pro Plus (the data are represented as the means ± SEM; *P < 0.05; unpaired, two-tailed Student’s *t* test). **h** Cellular fractionations of colons from the UC patients we collected were analyzed by immunoblotting with the indicated antibodies (*n* = 10)

To determine whether intestinal GOLM1 depletion altered the epithelial lineage commitment in IECs, we examined each cellular lineage of the absorptive or secretory lineages by immunohistochemical staining. Compared with *Golm1*^*−flox/flox*^ counterparts, there were more colonocytes and fewer enteroendocrine cells in untreated *Golm1*^*−ΔIEC*^ mice colons (Fig. 5a). We then examined four major signaling pathways involved in regulating IEC differentiation and fate determination in *Golm1*^*−ΔIEC*^ and *Golm1*^*−flox/flox*^ mice.^27^ While no significant change was detected in the key genes of the Wnt/β-catenin, TGF-β/BMP and Hedgehog pathways, the key genes of Notch pathway were markedly increased in IECs from *Golm1*^*−ΔIEC*^ mice compared to those from *Golm1*^*−flox/flox*^ mice (Fig. 5b). Furthermore, in CRC and IBD patient cohorts, *GOLM1* expression was negatively correlated with the expression of *HES/HEY* family genes which belong to Notch signaling downstream genes (Supplementary Fig. 5a–d). These findings suggested the inverse relevance of GOLM1 expression and Notch signaling activation.

As only NOTCH1 and NOTCH2 have been implicated in intestinal barrier maintenance,^28^ we analyzed nuclear translocation of NOTCH1 and NOTCH2 intracellular domains (NICD and N2ICD) in the absence of GOLM1. Even though Notch signaling downstream genes were upregulated in GOLM1 deficient mice, the NICD nuclear-to-cytoplasm ratio exhibited no significant change, suggesting that altered Notch signaling led by GOLM1 depletion did not relate to NOTCH1 activation (Fig. 5c). We then revealed that GOLM1 deficient IECs exhibited much more nuclear translocation of N2ICD, while most of N2ICD located in the cytoplasm in *Golm1*^*−flox/flox*^ IECs (Fig. 5c and Supplementary Fig. 5e). Similarly, *Golm1* silencing in human epithelial CRC cell line-Caco-2 upregulated Notch downstream genes and enhanced N2ICD nuclear translocation while GOLM1 overexpression had opposite effects (Fig. 5d–f and Supplementary Fig. 5f). Furthermore, we knocked down NICD or N2ICD expression in Caco-2 cells and then overexpressed *Golm1* (Supplementary Fig. 5g). Compared with individual Notch receptor depletion, simultaneous knockdown of NICD expression with *Golm1* overexpression led to a greater reduction in *Hes1* expression; however, simultaneous knockdown of N2ICD expression and overexpression of *Golm1* failed to cause further reduction in *Hes1* and *Hes5* expression, indicating that *Golm1* and N2ICD regulated Notch downstream signaling in the same pathway (Supplementary Fig. 5h). To confirm a causative link between low GOLM1 expression and nuclear N2ICD in human biopsies, we examined 30 CRC samples in tissue microarray by immunohistochemistry staining. We observed that GOLM1 expression levels were significantly lower in tumoral tissues than in peri-tumoral tissues; and nuclear staining of N2ICD appeared in nearly 20% tumoral samples while it appeared much less in peri-tumoral tissues (Fig. 5g). Furthermore, we observed similar inverse relevance of GOLM1 expression and Notch2 activation in IBD patients by immunoblotting (Fig. 5h). These data suggest that GOLM1 negatively modulates Notch pathway by sequestering N2ICD in the cytoplasm.

### GOLM1 interacts with N2ICD to modulate Notch downstream signaling

**Fig. 6:**
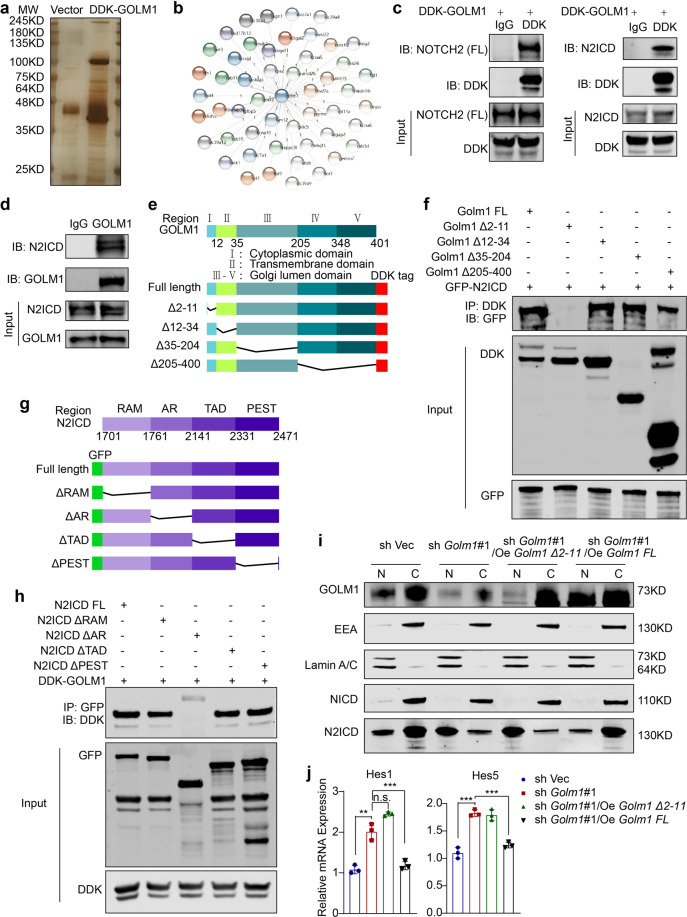
GOLM1 interacts with N2ICD and modulates its downstream signaling. **a** Mass spectrometry (MS) analysis of GOLM1-associated proteins. Total cell lysates from DDK-GOLM1 expressed cells were subjected to affinity purification. The purified protein complex was resolved on SDS-PAGE and silver stained; then the bands were retrieved and analyzed by MS. **b** A diagram depicts GOLM1 interactors as detected by MS (Detailed information in Supplementary Information). **c** The interaction between GOLM1 and NOTCH2/N2ICD in 293T cells stably expressing DDK GOLM1 was detected by immunoprecipitation. **d** The interaction between GOLM1 and NOTCH2 (FL)/N2ICD in Caco-2 cells was detected by immunoprecipitation. **e** Diagrammatic representation of GOLM1 and its truncated forms. Based on sequence and structure analyses, cytoplasmic domain, transmembrane domain, and Golgi lumen domain are indicated. **f** Various GOLM1 truncation constructs tagged with DDK were co-transfected with GFP-NOTCH2 in 293T cells for domain mapping. Immunoprecipitation analysis was performed with anti-GFP or anti-DDK antibodies. **g** Diagrammatic representation of N2ICD and its truncated forms. **h** Various N2ICD truncation constructs tagged with GFP were co-transfected with DDK-GOLM1 in 293T cells for domain mapping. Immunoprecipitation analysis was performed with anti-GFP or anti-DDK antibodies. **i** Cellular fractionations from GOLM1-deficient Caco-2 cells transfected with various GOLM1 truncation constructs and control cells were analyzed by immunoblotting with the indicated antibodies. **j** The relative mRNA expression levels of Notch signaling downstream genes from GOLM1-deficient Caco-2 cells transfected with various GOLM1 truncation constructs and control cells were determined by qRT-PCR (the data are represented as the means ± SEM; **P < 0.01, ***P < 0.001; unpaired, two-tailed Student’s *t* test)

To further elucidate the molecular mechanisms underlying defective GOLM1-mediated Notch signaling overactivation, GOLM1 interactors were identified by immunoaffinity purification and subsequent high-throughput mass spectrometry (LC-MS/MS) (Fig. 6a). NOTCH2 was recognized as one of the potential interactors (Fig. 6b and Supplementary Table 4). The interaction of GOLM1 with NOTCH2 was verified by co-immunoprecipitation. Both exogenous and endogenous GOLM1 interacted with full-length NOTCH2 and cleaved N2ICD (Fig. 6c, d). The colocalization of GOLM1 and N2ICD in the cytoplasm also supported this interaction (Supplementary Fig. 6a). Moreover, we mapped the regions of GOLM1 and N2ICD responsible for their interaction. A series of constructs encoding different GOLM1 truncates fused with MYC-DDK tags were transfected into 293T cells (Fig. 6e). Almost all GOLM1 fragments, except Δ2–11, were able to bind to N2ICD, suggesting that the cytoplasmic domain of GOLM1 binds to N2ICD (Fig. 6f). Likewise, through domain mapping of N2ICD, we demonstrated that the ankyrin repeat domain of NOTCH2 which mediates protein-protein interactions was essential and sufficient for its association with GOLM1 (Fig. 6g, h).

To determine whether GOLM1 depletion-mediated N2ICD nuclear translocation is dependent on GOLM1-N2ICD interaction, we co-transfected exogenous truncated and full-length GOLM into GOLM1 deficient Caco-2 cells. Re-introduction of the GOLM1 truncate (Δ2–11) failed to suppress excessive NOTCH2 nuclear translocation and decrease Notch downstream gene as full-length GOLM1 overexpression did (Fig. 6i, j). These results demonstrate that the GOLM1-NOTCH2 interaction is crucial for maintaining Notch signaling equilibrium in IECs.

### Notch inhibitor alleviates DSS-induced colitis and pro-tumorigenic inflammation via restoring intestinal barrier function in *Golm1*^*−ΔIEC*^ mice

**Fig. 7:**
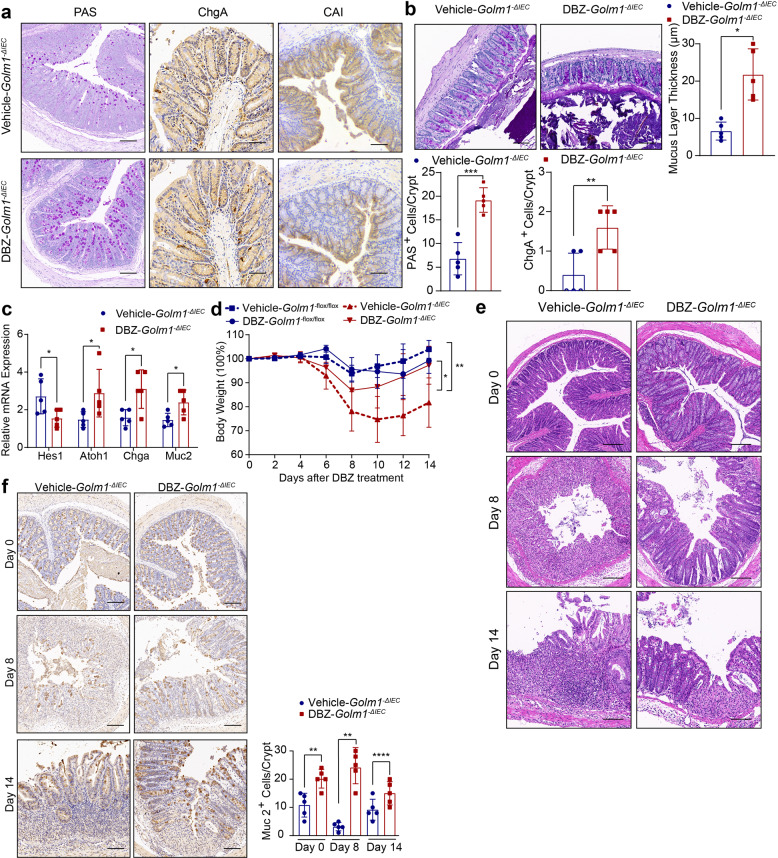
Notch inhibitor-DBZ treatment reduces DSS-induced colitis in *Golm1*^−*ΔIEC*^ mice. **a** Representative PAS, ChgA and CAI staining of mouse colon tissues obtained from *Golm1*^*−ΔIEC*^ mice injected intraperitoneally with 3 μmol/kg DBZ or vehicle for 5 days. Scale bars, 100 μm. Quantifications of PAS and ChgA staining are shown in the histogram (the data are represented as the means ± SEM, *n* = 5; **P < 0.01, ***P < 0.001; unpaired, two-tailed Student’s *t* test). **b** Representative PAS staining of mouse colon sections fixed by Carnoy’s fluid from *Golm1*^*−ΔIEC*^ mice injected intraperitoneally with 3 μmol/kg DBZ or vehicle for 5 days. Scale bars, 50μm. Quantification is shown in the histogram (the data are represented as the means ± SEM, *n* = 5; **P < 0.01; unpaired, two-tailed Student’s *t* test). **c** The relative mRNA expression levels of the indicated genes in the distal colon of mice determined by qRT-PCR after DBZ/vehicle treatment. The relative expression reflects the fold change calculated by comparing with the expression levels in untreated *Golm1*^*−ΔIEC*^ mice (the data are represented as the means ± SEM, *n* = 5; *P < 0.05; unpaired, two-tailed Student’s *t* test). **d** The body weights of *Golm1*^*−flox/flox*^ and *Golm1*^*−ΔIEC*^ mice treated with 2% DSS followed by DBZ administration were recorded on indicated days (the data are represented as the means ± SEM, *n* = 5; *P < 0.05, **P < 0.01). **e** Representative H&E staining of mouse colon sections obtained from DBZ/vehicle-DSS-treated *Golm1*^*−ΔIEC*^ mice sacrificed on indicated days. Scale bars, 100 μm. **f**. Representative MUC-2 staining of mouse colon sections obtained from *Golm1*^*−ΔIEC*^ mice treated with 2% DSS plus DBZ and sacrificed on indicated days. Scale bars, 100μm. Quantification is shown in the histogram (the data are represented as the means ± SEM, *n* = 5; **P < 0.01, ***P < 0.001; unpaired, two-tailed Student’s *t* test)

Since GOLM1 deficiency led to robust activation of Notch signaling, we assessed whether blocking Notch signaling could rescue the secretory lineage differentiation defects in *Golm1*^*−ΔIEC*^ mice. PAS^+^ goblet cells, ChgA^+^ enteroendocrine cells, and mucus layer thickness were increased and CAI^+^ colonocytes were decreased in Notch inhibitor-DBZ-treated *Golm1*^*−ΔIEC*^ mice compared to untreated *Golm1*^*−ΔIEC*^ mice (Fig. 7a, b). In line with this, DBZ-treated *Golm1*^*−ΔIEC*^ mice showed downregulated *Hes1* and upregulated expression of secretory lineage makers (Fig. 7c). Moreover, DBZ administration before DSS insultation alleviated colitis-related body weight reduction and epithelial damage in *Golm1*^*−ΔIEC*^ mice (Supplementary Fig. 7a and Fig. 7d, e). Compared with *Golm1*^*−ΔIEC*^ mice without DBZ, elevated MUC-2 production and normal-appearing crypts were observed during the recovery phase of DBZ/DSS-treated *Golm1*^*−ΔIEC*^ mice (Fig. 7f). DBZ also directed IEC secretory lineage differentiation in *Golm1*^*−flox/flox*^ mice (Supplementary Fig. 7b, c). However, the restrained body weight gain suggested a delayed recovery of DBZ-treated *Golm1*^*−flox/flox*^ mice after DSS insultation (Fig. 7d). The different effects of DBZ on DSS-treated *Golm1*^*−ΔIEC*^ and *Golm1*^*−flox/flox*^ mice stemmed from their differed basal states of Notch signaling activation, highlighting the equilibrium of Notch signaling in maintaining intestinal homeostasis.

## Discussion

The role of GOLM1 in CRC has not been investigated previously. Through analyzing the clinical IBD and CRC patients’ samples, we found that GOLM1 expression was decreased in colitis and cancerous tissues compared with normal colonic tissues. Systematic and IEC-specific depletion of GOLM1 conferred mice susceptible to DSS-induced colitis and AOM/DSS-induced CAC. GOLM1 knockout in IECs disrupted intestinal homeostasis and aggravated intestinal disorders via activating excessive Notch signaling. Blockade of Notch pathway rescued the impaired intestinal barrier integrity and robust colitis in GOLM1 deficient mice. Therefore, GOLM1 involves in maintaining intestinal homeostasis and suppressing colon cancer carcinogenesis.

In tissues with low basal GOLM1 expression such as liver, lung, and breast, aberrantly elevated GOLM1 has been reported to participate in oncogenic signaling using xenograft models.^15,29^ Identification of GOLM1 function in cancer could be limited due to the lack of precise genetic models which can be used to systematically determine GOLM1’s role in situ. Extrapolation of GOLM1 function in cancer from these models should be approached with caution. Cancer progression requires the orchestration between cell-autonomous signaling and tumor microenvironment.^30^ The role of GOLM1 in cancer may tissue-specific and context dependent. The basal expression of GOLM1 in colon is high. According to our TCGA dataset analysis, GOLM1 expression is significantly lower in colon tumor tissues and is associated with poor prognosis of CRC patients. We revealed that GOLM1 suppresses tumorigenesis during the onset of CRC by maintaining intestinal barrier integrity and alleviating pro-tumorigenic inflammation. As CRC develops, the effects of GOLM1 may rely on the orchestration between its oncogenic impact and intestinal homeostatic role. The definite function of GOLM1 in different stages of CRC needs to be unraveled through further investigation.

In order to investigate how GOLM1 deletion in IECs specifically contribute to intestinal barrier breakdown, we dissected the major determinants of intestinal homeostasis and found altered IEC differentiation and repressed secretory cell lineage in *Golm1*^*−ΔIEC*^ mice. Of particular importance is the reduction of mucus-producing goblet cells, a major risk factor for intestinal inflammation. We not only observed decreased number of goblet cells but also found defective goblet cell development and maturation in *Golm1*^*−ΔIEC*^ mice. The goblet cell markers and terminal maturation effectors (*Muc2, Spdef, and Klf4*) were also markedly downregulated due to GOLM1 depletion in IECs. It should be noted that mice deficient in either of MUC-2, KLF4, and SPDEF exhibit similar goblet cell deficiency and enhanced sensitivity to DSS-induced colitis and colon tumor formation.^13,31,32^ We also found significant changes in the expression of transcription factors controlling the upstream effectors of IEC fate decisions. Increased *Hes1* expression and correspondingly decreased *Atoh1* expression indicate hyperactive Notch signaling, which is consistent with excessive enterocytes proliferation at the expense of the secretory cell lineage in *Golm1*^*−ΔIEC*^ mouse intestines.^33,34^ *Rosa-Notch/Cre* + mice, which express sustained Notch signaling activation in the intestinal epithelium, exhibit similar phenotypes.^35^ However, Notch inactivation via genetically deleting the principle component of Notch signaling such as *Rpb-j* or *Pofu1* also impaired IEC differentiation and intestinal barrier integrity, indicating neither outright inactivation nor overactivation of Notch signaling was desired for intestinal homeostasis.^36,37^ As only Notch1 and Notch2 have been implicated in intestinal barrier maintenance, we suggest that hyperactive Notch2 may contribute to the GOLM1 depletion-mediated defects since Notch2 nuclear-to-cytoplasm ratio was altered instead of Notch1 in GOLM1 deficient mice.^38^ Yet more genetic evidence is required to further delineate whether activated Notch2 is the main driver of the defective phenotypes in GOLM1 deficient mice.

The role of Notch signaling is complicated in CRC tumorigenesis, progression, and metastasis, which acts either oncogenically or suppressively with multiple cell-autonomous and non–cell-autonomous mechanisms. Although mice lacking NOTCH1 in IECs develop spontaneous CRC, Notch signaling activation is present in clinical CRC samples and is essential for the self-renewal of tumor-initiating cells and the development of adenomas in *ApcMin/+* mice.^39–41^ Its potential to increase proliferation and survival of intestinal stem cells provides another possible explanation for the susceptibility of GOLM1 deficient mice to CRC.^35,42^ Furthermore, Notch signaling promotes CRC metastasis via creating a metastatic niche with vasculature and immune infiltration or modulating epithelial-mesenchymal transition.^43,44^ Its inhibition suppresses cell proliferation and tumor growth in preclinical models, prompting the usage of Notch signaling inhibitors for CRC in clinical trials.^45,46^ The impact of Notch signaling may be context dependent.

In summary, by revealing the role of GOLM1 in maintaining intestinal homeostasis to restrict colitis and colonic tumorigenesis, we demonstrate that GOLM1 exerts suppressive function on carcinogenesis distinct from its previously recognized oncogenic effect on liver, prostate, and lung malignancies. Of clinical relevance is that GOLM1 deficiency-mediated robust colitis, pro-tumorigenic inflammation, and CAC can be alleviated by either probiotic agents, mucosal safeguard or Notch signaling blockade.

## Materials and methods

### Mice

*Golm1*^*−/−*^ mice were generated as previously described.^15^ Epithelial *Golm1* knockout mice-*Golm1*^*−flox/flox,Cre(+)*^, referred as *Golm1*^*−ΔIEC*^ mice, were generated by crossing *Golm1*^*−flox/flox*^ mice with *Villin-cre* mice (Jackson Laboratory, Bar Harbor, ME). *GP73*^*−flox/flox, Cre(−)*^ mice, termed as wild type (*Golm1*^*−flox/flox*^) mice, were used as controls in all experiments. All mice were generated on a C57BL/6J background and maintained in a pathogen-free facility. All animal protocols were approved by the Animal Care and Use Committee of Peking Union Medical College.

### Human CRC and UC sample analysis

For the analysis of the GOLM1 expression in CRC and UC, human CRC samples (*n* = 10), UC samples (*n* = 10) with adjacent normal colon tissue were freshly obtained from patients undergoing surgery at the first medical center of PLA General Hospital. The patients’ information was included in Supplementary Table S1. The institutional review board at the first medical center of PLA General Hospital approved the study protocol, and all patients provided written informed consent. CRC and UC were confirmed after surgery by pathologists. Tissues were snap frozen and later were sonicated for immunoblotting. Tissue microarray slide (Cat.1401) is purchased from Servicebio (Wuhan, China).

### Cells and reagents

Caco-2 and 293T cell lines were purchased from ATCC (Manassas, VA). Cells were cultured in Dulbecco’s modified Eagle medium or Roswell Park Memorial Institute-1640, supplemented with 10% fetal bovine serum and 1% antibiotics in 5% CO_2_ at 37 °C unless described otherwise. DSS (mol wt. 36–40 KDa) was from Affymetrix (Santa Clara, CA, USA); AOM (#A2853) was from Sigma (St. Louis, MO, USA); VSL#3 was from CD Pharma India Pvt. Ltd; and DBZ(YO-01027) was from Selleck (Houston, TX, USA). Phosphate Buffered Saline was used as vehicle to dissolve the VSL#3. Rebamipde (HY-B0360)was from Selleck.

### Colitis and CAC model

For short-term colitis model establishment, mice (6–8 weeks old) were administered with 2% DSS for five consecutive days followed by regular drinking water for the rest of the days and sacrificed at indicated time points. For CAC model induction, the combination of carcinogen AOM and repeated DSS treatments were scheduled as below: mice (8–10 weeks old) were firstly injected intraperitoneally with a single dose of AOM (12.5 mg/kg), followed by three cycles of DSS treatment.

### H&E and immunochemistry staining

In brief, fresh colon tissues were fixed in 4% paraformaldehyde at 4 °C overnight, then subjected to paraffin imbedding sections. For H&E Staining, the sections were stained with haematoxylin and dehydration in graded alcohols and xylene. For immunolabeling, the sections were incubated with indicated primary antibodies: anti-CD3 [1:400, 85061; Cell Signaling]; anti-Ki67 [1:200, GB13030-2; Servicebio], anti-F4/80 [1:200, GB11027; Servicebio], anti-ChgA [1:400, ab45179; Cell Signaling] anti-CAI [1:200, SC39349; Santa Cruz], anti-Lysozyme [1:300, ab108502; Abcam], anti-N2ICD [1:200, YC0069; Immunoway], overnight at 4 °C in the dark. Then, the sections were incubated with either HRP–conjugated Goat anti-Rabbit IgG (1:200, G1215; Servicebio) or HRP–conjugated Goat anti-Mouse IgG (1:200, G1214; Servicebio) for 50 min at 25 °C. The subsequent detection was performed using the standard substrate detection of DAB. TUNEL assay kit was purchased from Abcam (ab66110). Images were taken by using Leica DM6 B Upright Microscope.

### Immunofluorescence staining

Paraffin sections of colon tissues were firstly dehydrated in gradient ethanol and blocked by 5% BSA in 0.2% TritonX-100/PBS, then incubated with specific primary antibody overnight at 4 °C: anti-GP73 [1:100, sc-48011; Santa Cruz],anti-N2ICD [1:100, YC0069; Immunoway], followed by incubation of secondary antibodies accordingly: Cy3 conjugated Goat Anti-Rabbit IgG (H+L) [1:300, GB21303; Servicebio] or FITC conjugated Donkey Anti-Goat IgG (H+L)[1:200, GB22404; Servicebio], and preserved in mounting medium with DAPI. The fluorescence was analyzed using Olympus Confocal Microscope.

### Immunoblotting

Cells were lysed by lysis buffer (2% sodium dodecyl sulfate, 10% glycerol, 10 mmol/L Tris, pH 6.8, and 100 mmol/L dithiothreitol) with protease inhibitor cocktail, and the colonic tissues were grinded in a homogenizer (KZ-II, Servicebio) following the manufacturer’s instructions with lysis buffer. The Extracts from cells or tissues were boiled for 10–15 min and subjected to SDS-PAGE electrophoresis. The immunoblotting was performed as previously described.^47^ Antibodies were listed in Supplemental Materials Table S2.

### Disease activity index (DAI) assessment

DAI was determined by combining scores of a) weight loss b) stool consistency and c) bleeding status. Each score was determined as follows: changes in weight loss (0:<1%, 1: 1–5%, 2: 5–10%, 4:>15%); stool consistency (0: normal, 2: loose stools, 4: diarrhea); stool blood (0: negative, 2: positive) or gross bleeding (4).

### Histological analysis of epithelial damage

Histological analysis of epithelial damage after DSS treatment was scored as follows (modified from^48^): Intact crypt (damage score 0); loss of basal 1/3 of the crypt (damage score 1). loss of the entire crypt but intact surface epithelial cells (damage score 3); loss of both the entire crypt and the surface epithelial cells (damage score 4).

### Real-time quantitative PCR

Total RNA was extracted from cells or colonic tissues using Trizol (Invitrogen) under the manufacturer’s instructions. First strand cDNA was synthesized using the PrimeScript RT Reagent Kit (Takara). Real-time PCR was performed using TransStart Green quantitative polymerase chain reaction SuperMix (TransGen Biotech, Beijing, China) to quantify the expression of mRNA. Primers were designed on exon junctions to prevent co-amplification of genomic complementary DNA, and the sequences were listed in Supplemental Materials Table S3.

### Bone marrow chimera transplantation

CD45.2-expressing *Golm1*^*−flox/flox*^ and CD45.2-expressing *Golm1*^*−/−*^ mice were lethally irradiated with a single dose of 1000 *rads*. Irradiated mice were adoptively transferred with 5×10^6^ bone marrow cells collected from CD45.1-expressing *Golm1*^*−flox/flox*^/ CD45.2-expressing *Golm1*^*−flox/flox*^ and CD45.2-expressing *Golm1*^*−/−*^ mice respectively in the next day. Mice were maintained on sulfamethoxazole and trimethoprim (Bactrim) antibiotics diluted in drinking water for 5 weeks after reconstitution. The following four groups of chimera mice were generated: *Golm1*^*−flox/flox*^/*Golm1*^*−flox/flox*^ mice (CD45.1-expressing *Golm1*^*−flox/flox*^ cells into CD45.2-expressing *Golm1*^*−flox/flox*^ mice), *Golm1*^*−flox/flox*^/*Golm1*^*−/−*^mice (CD45.1-expressing *Golm1*^*−flox/flox*^ cells into CD45.2-expressing *Golm1*^*−/−*^ mice), *Golm1*^*−/−*^/*Golm1*^*−flox/flox*^ mice: (CD45.2-expressing *Golm1*^*−/−*^ mice into CD45.2-expressing *Golm1*^*−flox/flox*^ mice) and *Golm1*^*−/−*^/*Golm1*^*−/−*^ mice (CD45.2-expressing *Golm1*^*−/−*^ cells into CD45.2-expressing *Golm1*^*−/−*^ mice). The reconstitution rate was evaluated eight weeks after bone marrow transfer and determined by flow staining of CD45.1 and CD45.2 in mouse blood leukocytes.

### Electron microscopy

Pieces of intestine tissues were pre-fixed overnight at 4 °C in 2% paraformaldehyde and 2.5% glutaraldehyde in PBS, and then fixed in 2% OsO_4_ for 2 h at room temperature. After fixation, the tissue samples were dehydrated through gradient acetone, then progressively embedded in Epon epoxy resin. Sections with a thickness of 50 nm were cut with an ultramicrotome UCT6 (Leica Microsystems, Vienna) and placed on TEM grids (Formvarcarbon-coated Cu grids). The grids were further contrasted with uranyl acetate and lead citrate. Micrographs were obtained with a Jeol JEM 1400 plus electron microscope (Jeol, USA) operating at 80 kV.

### Intestinal Permeability

Mice were administered with FITC-dextran (Sigma #FD4) by oral gavage (44 mg/100 g body weight). Blood was collected by cardiac puncture from anesthetized mice 4 h later. The concentration of FITC in serum was determined by SynergyH1 automatic microplate reader (Biotek), in an excitation of 485 nm and an emission wavelength of 528 nm, and was normalized using serially diluted FITC-dextran standard.

### Intestinal epithelial cell isolation

Colon tissues dissected from the sacrificed mice were opened longitudinally and washed extensively in cold PBS. Colons were then cut into 3 mm^2^ pieces and incubated in 5 mM EDTA solution in 15 ml PBS on a shaker at 200 RPM for 25 min at room temperature. The supernatant was collected and then the above process was repeated once. Supernatants from the previous procedure were combined and centrifuged at 500 *g* for 8 min. The pelleted cells were isolated IECs. To separate cell nucleus and cytoplasm, isolated IECs were later subjected to nuclear and cytoplasmic extraction using NE-PER™ Nuclear and Cytoplasmic Extraction Reagents Kit (#78833, Thermo Scientific™).

### Nuclear and cytoplasmic extraction

To separate cell nucleus and cytoplasm, cells were subjected to nuclear and cytoplasmic extraction using NE-PER™ Nuclear and Cytoplasmic Extraction Reagents Kit (#78833, Thermo Scientific™). GAPDH and EEA antibodies were used as cytoplasm loading control and Lamin A/C was used as nuclear loading control for cellular fractionation immunoblotting analysis.

### Overexpression constructs and ShRNA Knockdown

The plasmid of Golm1 tagged with Myc-DDK (RC200086) and its control (PS100001) were purchased from Origene Technologies (USA). Transfection was performed using Lipofectamine™ 2000 (#11668027, Invitrogen). ShRNA Knockdown for Human NOTCH1 (sc-36095-V), Notch 2 and their controls were purchased from Santa Cruz. ShRNA Knockdown lentiviral particles for Human Golm1 and their controls were synthesized by GENECHEM (Shanghai, China). The target sequences were as follows: GP73 knockdown #1: GCCAGTGCATCAATCAGATGA; GP73-knockdown #2: GCATCATCGTCTTGGGCTTCA. Human colonic epithelial cell line-Caco2 was infected with lentiviral particles. The infection rate was evaluated through the expression of green fluorescent protein after incubation with virus after 48 h.

### Immunoprecipitation

Cells were lysed in cell lysis buffer (RM00022, Abclonal) with Protease/Phosphatase Inhibitor Cocktail (#5872, CST). For the interaction of exogenous Golm1 tagged with Myc-DDK and NOTCH2/N2ICD, anti-DDK (1:200, TA50011-1, Origene) antibody and control IgG antibody were added separately to each aliquot, and samples were rotated with protein G magnetic beads (HY-K0204, MCE) at 4 °C overnight. For the interaction of endogenous Golm1 and NOTCH2/N2ICD, anti-Golm1 (1:150, 15089-1-AP; Proteintech) antibody and control IgG antibody were added separately to each aliquot and samples were rotated with protein G magnetic beads (HY-K0204, MCE) at 4 °C overnight. The Magbeads-Ab-Ag complex was washed for 5 times using lysis buffer for each wash. Magnetic separation was performed between each wash, and the final immunocomplex supernatants were then subjected to immunoblotting.

### Statistical analysis

Cohort data were downloaded from NCBI. R language and Bioconductor were used for background correction, normalization, calculation of gene expression, and annotation. Graphpad Prism V.8 was used for statistical analysis (San Diego, California, USA). Data are presented as means ± SEM. Unpaired two-tailed Student’s test was used for datasets including two independent groups (n.s., no significance; *p < 0.05, **p < 0.01, ***p < 0.001). All data are representative of at least three independent experiments.

## Supplementary information

Supplementary Materials 2

Supplementary Materials 1

## Data Availability

The data of mass spectrometry are provided on Supplementary Materials 2; other datasets or information analyzed during the current study are available from the corresponding authors on reasonable request.
