# Supplementary material for: GOLM1 restricts colitis and colon tumorigenesis by ensuring Notch signaling equilibrium in intestinal homeostasis
Source: Signal Transduct Target Ther. 2021 Apr 14;6:148. doi: 10.1038/s41392-021-00535-1 (PMC8044123; doi:10.1038/s41392-021-00535-1)
Supplement: Supplementary file 2 — Supplementary Materials 1 [file 41392_2021_535_MOESM2_ESM.docx]

Supplementary Materials 1 for

**GOLM1 restricts colitis and colon tumorigenesis by ensuring Notch signaling equilibrium in intestinal homeostasis**

Yang Pu^1*#^, Ya Song^1, 2*^, Mengdi Zhang^1^, Caifeng Long^1^, Jie Li^1^, Yanan Wang^1^, Yinzhe Xu^4^, Fei Pan^4^, Xinyu Zhang^1^, Yanan Xu^3^, Jianxin Cui^4^, Hongying Wang^5^, Yan Li^2^, Yong Zhao^3^, Di Jin^2#^, Hongbing Zhang^1#^

Correspondence to：Hongbing Zhang (e-mail: hbzhang@ibms.pumc.edu.cn); Di Jin (e-mail: jindi0801@126.com); Yang Pu (e-mail: puyang_py@163.com)

**This file includes:**

Figures. S1 to S7

Tables S1, S2 and S3

***
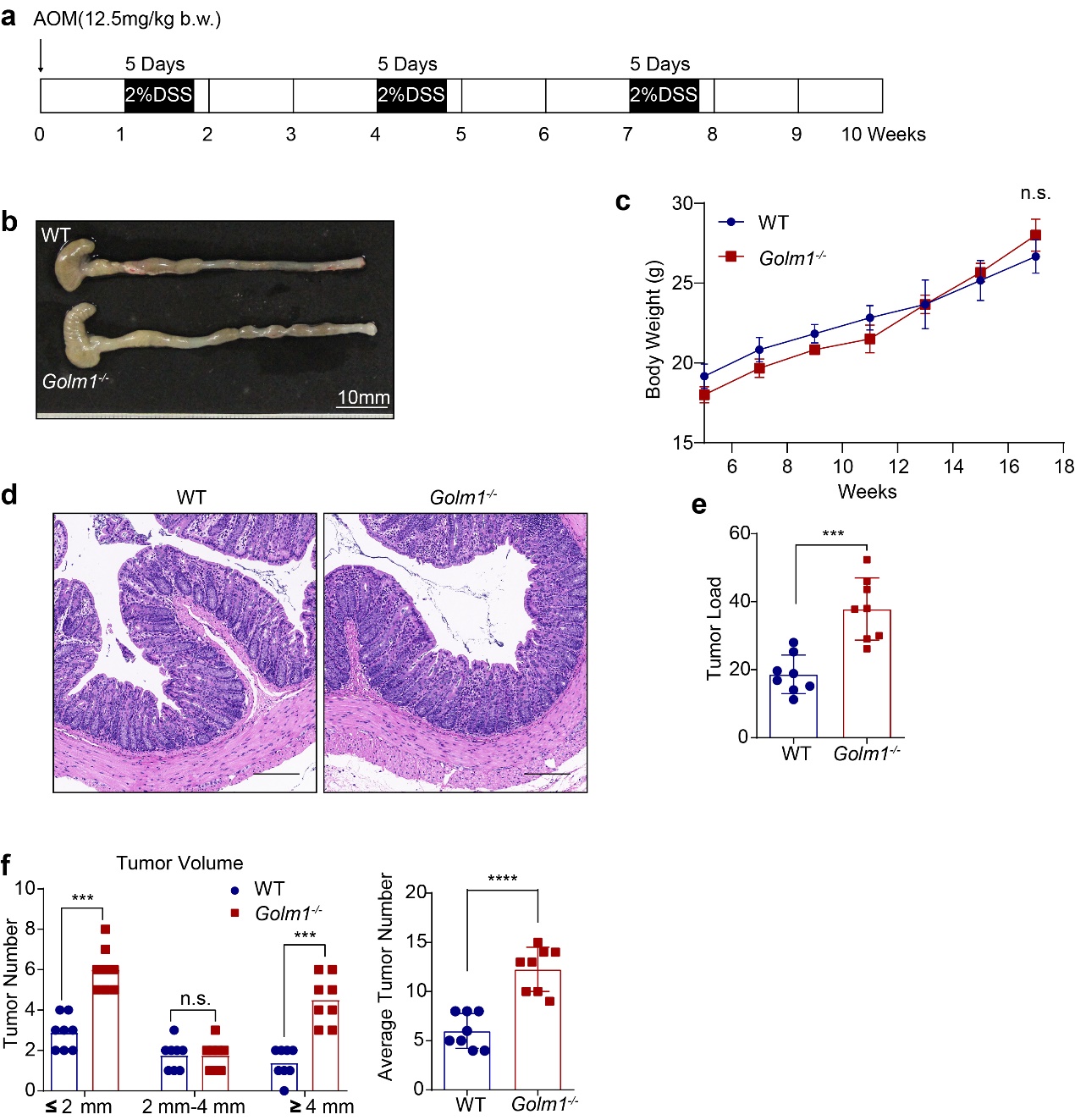
***

**Fig. S1 GOLM1 deficiency increases mouse susceptibility to AOM/DSS-induced colon tumorigenesis.**

1. A schematic representation of the AOM/DSS treatment protocol.
2. Representative image of colons obtained from WT and *Golm1^-/-^* mice.
3. The mouse body weights of WT and *Golm1^-/-^* mice were recorded on the indicated days (the data are represented as the means ± SEM, n=5).
4. Representative H&E staining of colon sections harvested from WT and *Golm1^-/-^* mice in steady-state. Scale bars, 100μm.
5. Average tumor loads of AOM/DSS-treated WT and *Golm1^-/-^* mice sacrificed on day 70 after AOM injection. The tumor load represents the tumor areas per mouse (the data are presented as the means ± SEM, n=8; ****P <* 0.001; unpaired, two-tailed Student’s t test).
6. The average colon tumor volume and number of AOM/DSS-treated mice (each symbol in each column represents an individual mouse in the left panel, n=8; the data are represented as the means ± SEM in the right panel, *****P <* 0.0001, ****P <* 0.001; unpaired, two-tailed Student’s t test).


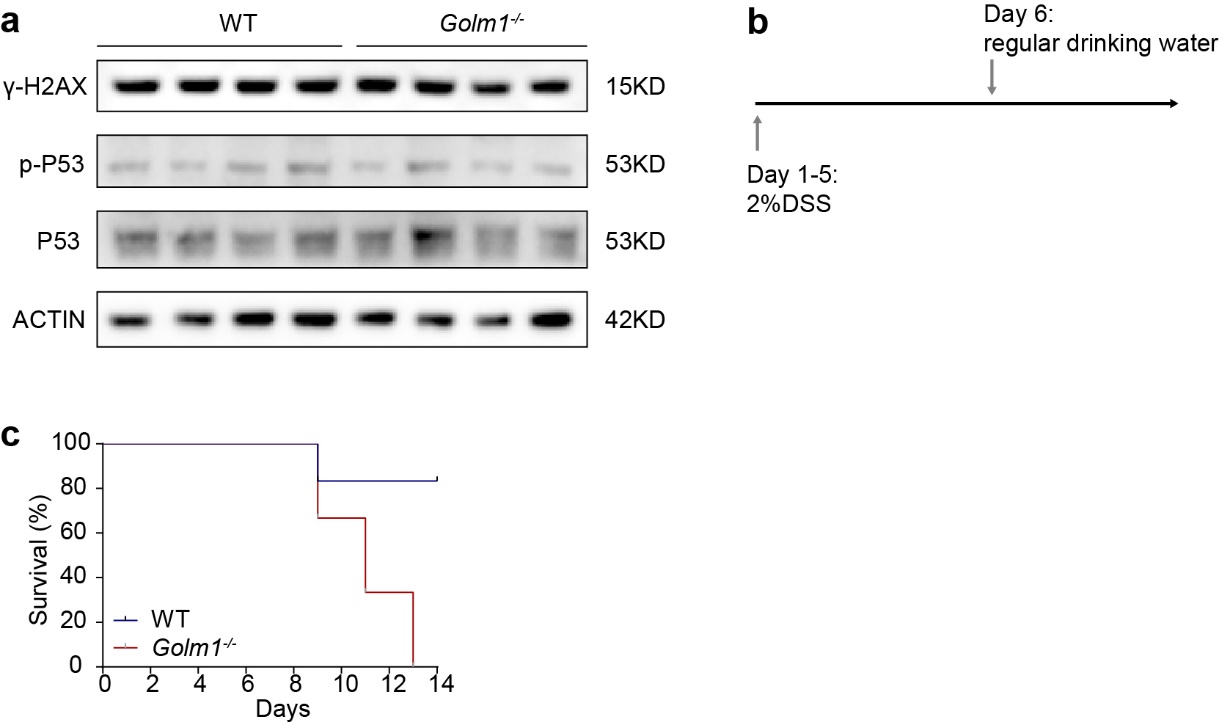


**Fig. S2 GOLM1 depletion renders mice more vulnerable to DSS-induced colitis.**

1. Colon lysates were prepared from AOM-treated mice and analyzed by immunoblotting (one mouse sample per lane, n=4) with the indicated antibodies.
2. A schematic representation of the DSS administration protocol.
3. Kaplan-Meier survival analysis of 3% DSS-treated *Golm1^-/-^* mice and WT counterparts (n=5; the P value was calculated by the log-rank test).

**
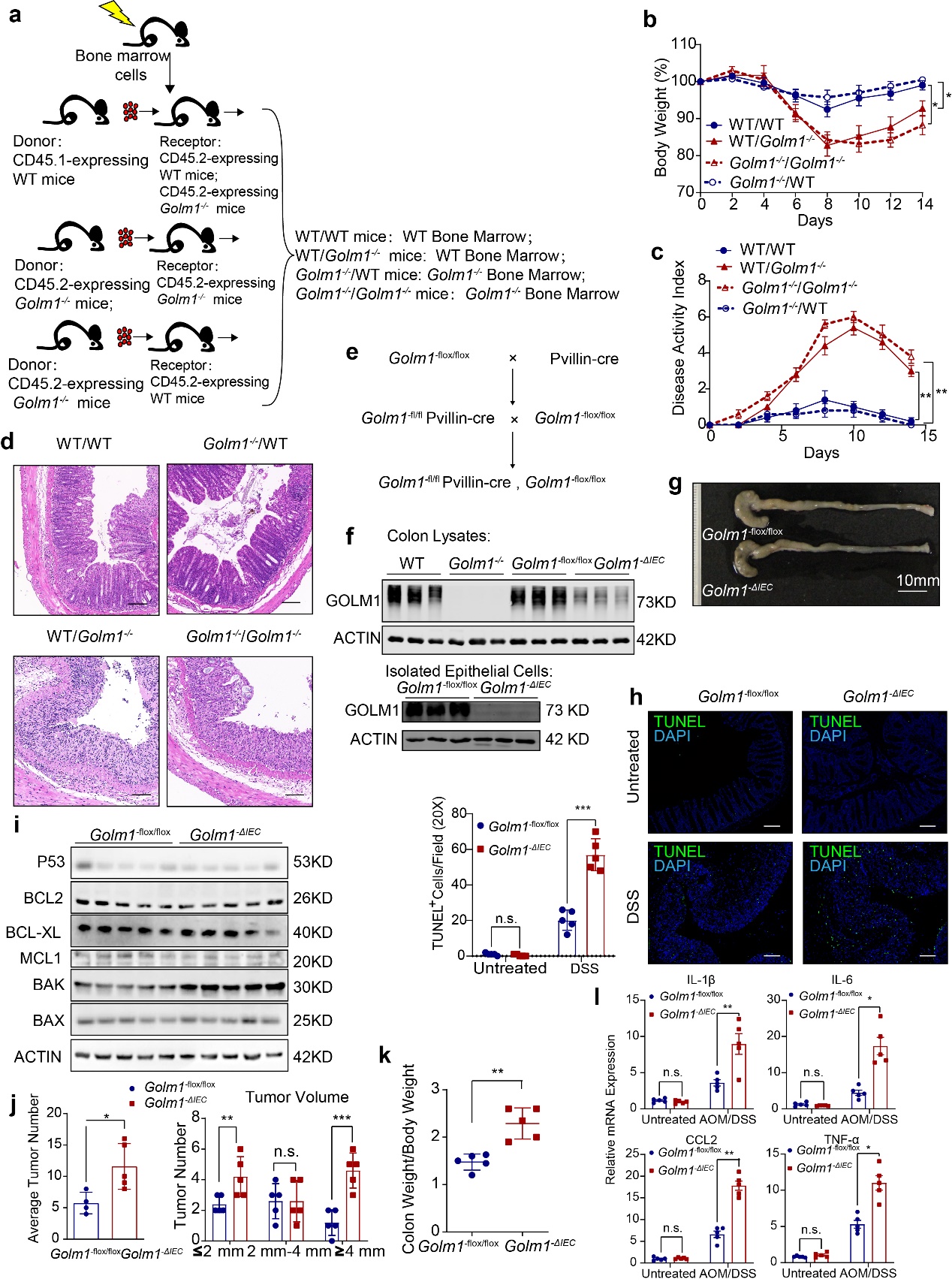
**

**Fig. S3 GOLM1 restrains DSS-induced colitis and CAC by modulating IECs.**

1. A schematic illustration of the strategy to establish bone marrow chimeras.
2. Bone marrow-transplanted (WT/WT, *Golm1^-/-^* /WT, WT/ *Golm1^-/-^*  and *Golm1^-/-^* /*Golm1^-/-^*) mice were administered 2% DSS in the drinking water for 5 days, and the mouse body weights were recorded on indicated days (the data are represented as the means ± SEM, n=5; **P <* 0.05).
3. The disease activity indexes of bone marrow-transplanted mice after 2% DSS administration for 5 days are shown (the data are represented as the means ± SEM, n=5; ***P <* 0.01).
4. Representative H&E staining of colon sections from the DSS-treated bone marrow-transplanted mice sacrificed on day 8. Scale bars, 100μm.
5. A schematic illustration of the strategy used to generate *Golm1^-△IEC^* mice.
6. The efficiency of GOLM1 knockout in whole-colon lysates or isolated epithelial cells from the indicated mice was analyzed by immunoblotting. (one mouse sample per lane, n=3).
7. Representative image of colons from untreated *Golm1^-△IEC^* mice and WT mice.
8. Representative TUNEL staining of colon sections obtained from 2% DSS -treated mice sacrificed on day 8. Scale bars, 100μm. Quantification is shown in the histogram. (the data are represented as the means ± SEM, n=5; ****P <* 0.0001; unpaired, two-tailed Student’s t test).
9. DSS-treated mice were sacrificed on day 8, and isolated epithelial cells were analyzed by immunoblotting (n=5) with the indicated antibodies.
10. The average colon tumor volume and number in AOM/DSS-treated mice (each symbol in each column represents an individual tumor in the right panel; the data are represented as the means ± SEM in the right panel, **P <* 0.05, ***P <* 0.01; unpaired, two-tailed Student’s t test).
11. The average ratio of colon weight/body weight in AOM/DSS-treated mice (each symbol in each column represents an individual mouse; the data are represented as the means ± SEM; n=5, ***P <* 0.01; unpaired, two-tailed Student’s t test).
12. Relative mRNA expression levels of inflammatory mediators in the distal colon of AOM/DSS-treated mice determined by qRT-PCR. Relative expression reflects the fold change calculated by comparing with the average expression levels in untreated *Golm1^-flox/flox^* (the data are represented as the means ± SEM, n=5; **P <* 0.05, ***P <* 0.01; unpaired, two-tailed Student’s t test).


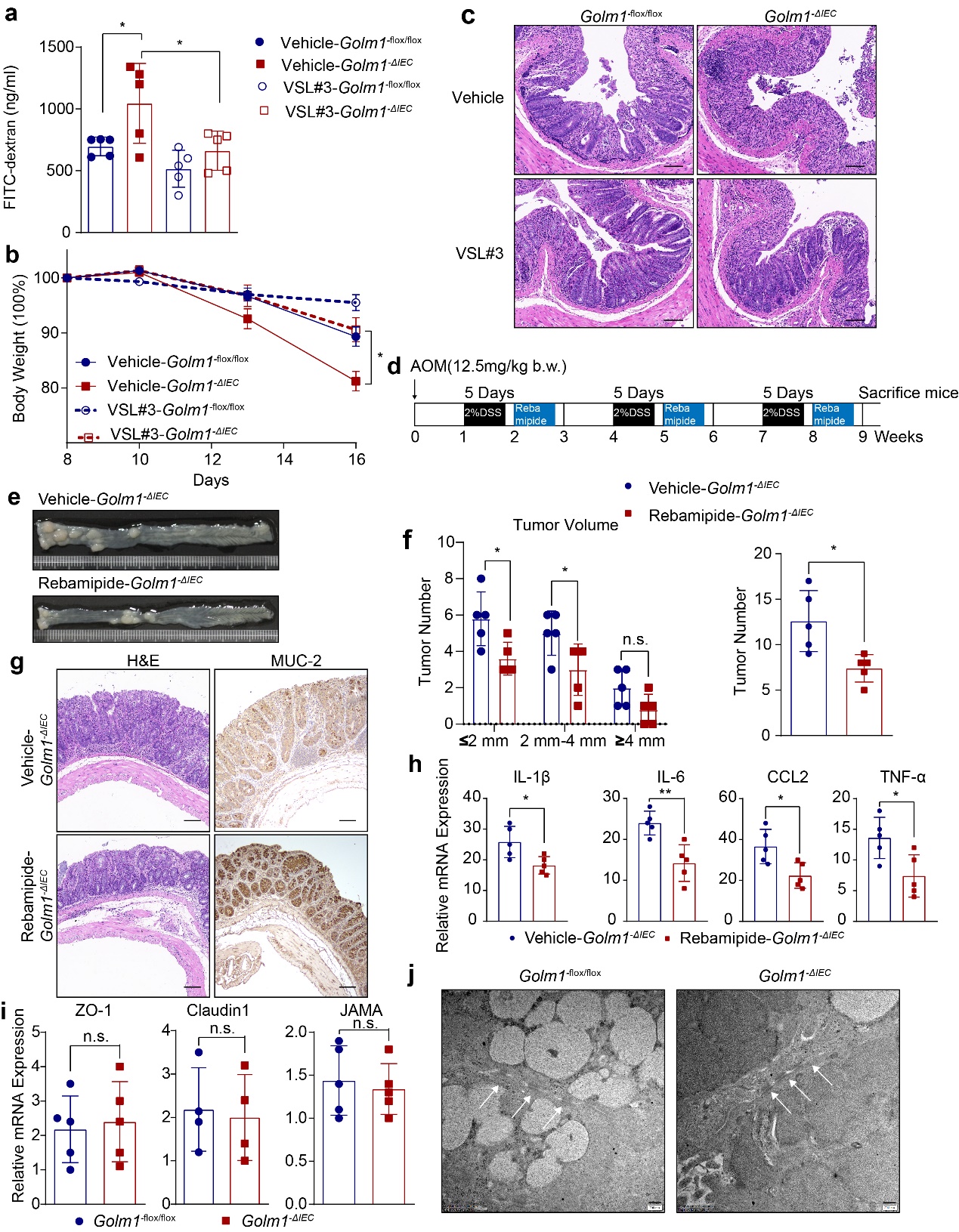


**Fig.S4 GOLM1 is required for IEC differentiation and proliferation.**

1. Intestinal permeability was determined after mice treated with vehicle or the probiotic mixture VSL#3 for 7 days (the data are represented as the means ± SEM, n=5; **P <* 0.05; unpaired, two-tailed Student’s t test).
2. Mice were administered with 2% DSS for 5 days followed by one week of vehicle/VSL#3 treatment. Mouse body weights were recorded on indicated days (the data are represented as the means ± SEM, n=5; **P <* 0.05).
3. Representative H&E-stained colon sections harvested from mice administered with VSL#3 plus DSS administration. Scale bars, 100μm.
4. A schematic representation of the AOM/DSS combined with rebamipide administration in *Golm1^-△IEC^* mice.
5. Representative images of colon tumors harvested from mice administered with AOM/DSS plus rebamipide.
6. The average colon tumor volume and number of AOM/DSS plus rebamipide treated mice (each symbol in each column represents an individual mouse in the left panel, n=5; the data are represented as the means ± SEM in the right panel, **P <* 0.05; unpaired, two-tailed Student’s t test).
7. Representative H&E and MUC-2 staining of colon sections obtained from AOM/DSS plus rebamipide treated mice. Scale bars, 50μm.
8. Relative mRNA expression levels of inflammatory mediators in the distal colon of AOM/DSS plus rebamipide treated mice determined by qRT-PCR. Relative expression reflects the fold change calculated by comparing with the average expression levels in untreated mice (the data are represented as the means ± SEM, n=5, **P <* 0.05, ***P <*0.01; unpaired, two-tailed Student’s t test).
9. Relative mRNA expression levels of the indicated genes in colons from untreated *Golm1^-△IEC^* and *Golm1^-flox/flox^* mice determined by qRT-PCR (the data are represented as the means ± SEM, n=5, unpaired, two-tailed Student’s t test).
10. Representative electron microscope images showing tight junctions in colon epithelia obtained from *Golm1^-△IEC^* and *Golm1^-flox/flox^* mice. Scale bars, 100nm. White arrows indicate kissing points of tight junction.


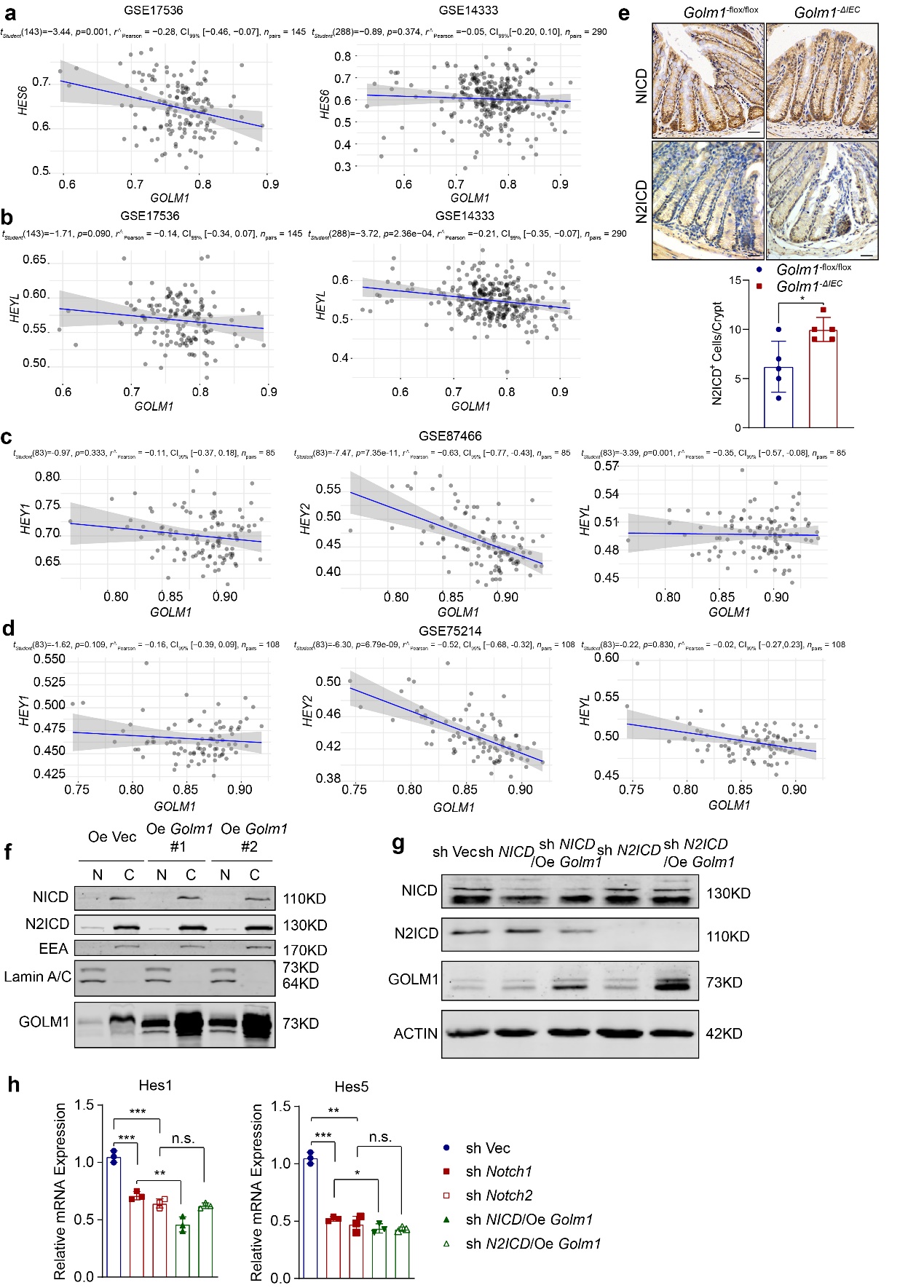


**Fig. S5 GOLM1 deletion leads to the nuclear translocation of N2ICD.**

1. Expression levels of *HES6* along with *GOLM1* were analyzed in CRC patients of cohort GSE17536 and cohort GSE14333.
2. Expression levels of *HEYL* along with *GOLM1* were analyzed in CRC patients of cohort GSE17536 and cohort GSE14333.
3. Expression levels of *HEY1, HEY2 and HEYL* along with *GOLM1* were analyzed in UC patients of cohort GSE87466.
4. Expression levels of *HEY1, HEY2 and HEYL* along with *GOLM1* were analyzed in UC patients of cohort GSE75214.
5. Representative NICD and N2ICD staining of colon sections from *Golm1^-△IEC^* and *Golm1^-flox/flox^* mice under steady-state conditions. Scale bars, 50μm. Quantification is shown in the histogram (the data are represented as the means ± SEM, n=5; **P <* 0.05; unpaired, two-tailed Student’s t test).
6. Cellular fractionations from *GOLM1* overexpressed-Caco-2 cells and control cells were analyzed by immunoblotting with the indicated antibodies.
7. GOLM1 overexpression in *NICD*-knockdown or *N2ICD*-knockdown Caco-2 cells was confirmed by Immunoblotting.
8. Relative mRNA expression levels of Notch downstream genes (*Hes1* and *Hes5*) in *NICD/N2ICD*-deficient Caco-2 cells with *GOLM1* overexpression were determined by qRT-PCR (the data are represented as the means ± SEM; **P <* 0.05, ***P <* 0.01, ****P <* 0.001; unpaired, two-tailed Student’s t test).

**
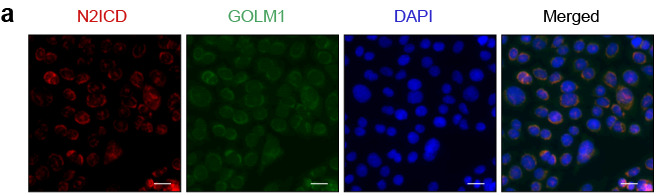
**

**Fig. S6 GOLM1 interacts with N2ICD.**

1. Immunofluorescence of GOLM1 and N2ICD co-localization in Caco-2 cells. The nuclei are counterstained with DAPI. Scale bars, 20μm.


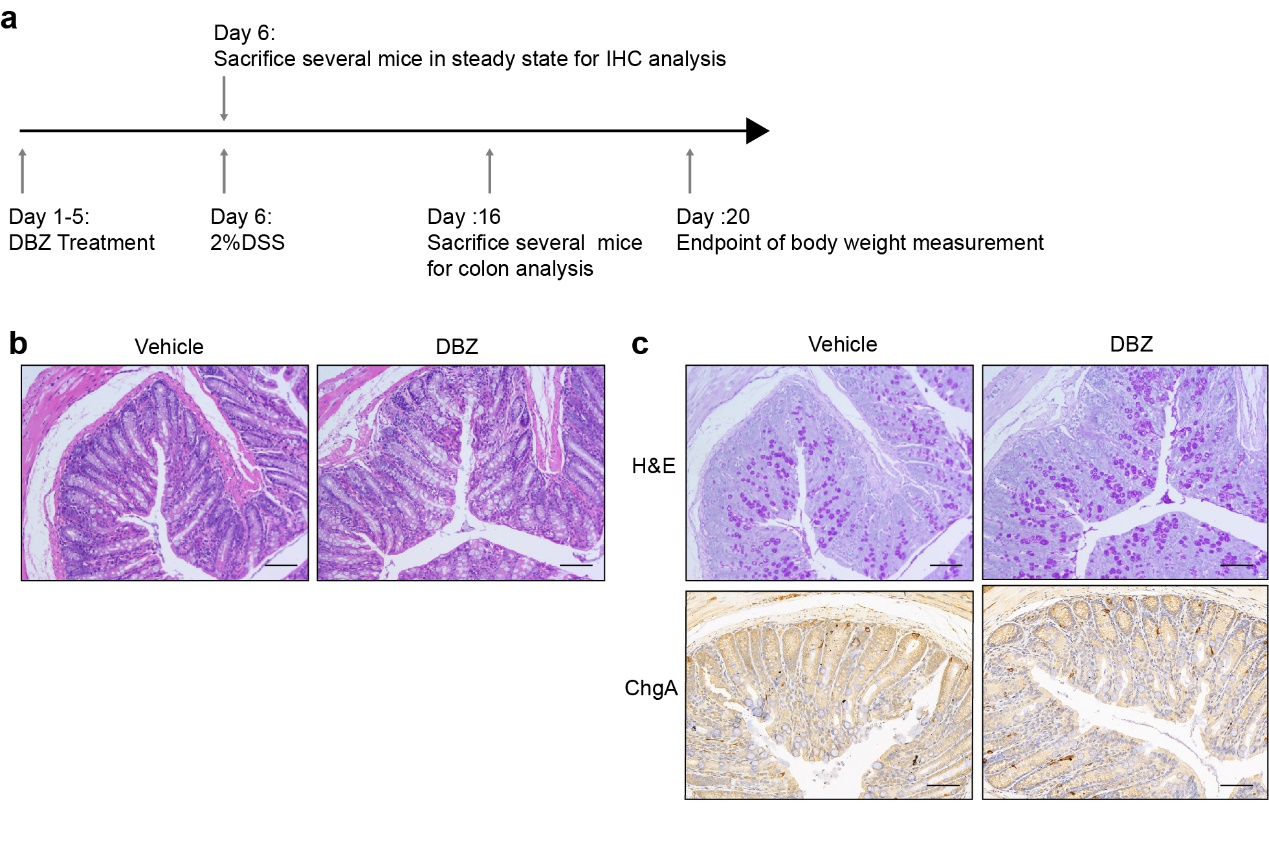


**Fig. S7 Notch inhibitor-DBZ treatment reduces the DSS-induced colitis in *Golm1^-△IEC^***

**mice.**

1. A schematic of the strategy to administer the Notch inhibitor and DSS.
2. Representative H&E staining of colon sections obtained from *Golm1^-flox/flox^* mice injected intraperitoneally with 3 μmol/kg DBZ or vehicle for 5 days. Scale bars, 100μm.
3. Representative PAS and ChgA staining of colon sections from *Golm1^-flox/flox^* mice injected intraperitoneally with 3 μmol/kg DBZ or vehicle for 5 days. Scale bars, 100μm.

Table S1. Clinical information of CRC and IBD patients.

| **CRC patients** | | | | |
| --- | --- | --- | --- | --- |
| No. | Age | Sex | Organ | Pathological Diagnosis |
| 1 | 65 | Male | Colon | Adenocarcinoma |
| 2 | 78 | Male | Colon | Adenocarcinoma |
| 3 | 46 | Male | Colon | Adenocarcinoma |
| 4 | 55 | Male | Colon | Adenocarcinoma |
| 5 | 80 | Male | Colon | Adenocarcinoma |
| 6 | 68 | Male | Colon | Adenocarcinoma |
| 7 | 72 | Male | Colon | Adenocarcinoma |
| 8 | 59 | Male | Colon | Adenocarcinoma |
| 9 | 63 | Male | Colon | Adenocarcinoma |
| 10 | 72 | Male | Colon | Adenocarcinoma |
| **IBD patients** | | | | |
| No. | Age | Sex | Organ | Pathological Diagnosis |
| 1 | 48 | Male | Colon | Ulcerative Colitis (UC) |
| 2 | 35 | Male | Colon | UC |
| 3 | 54 | Male | Colon | UC |
| 4 | 40 | Male | Colon | UC |
| 5 | 52 | Male | Colon | UC |
| 6 | 39 | Male | Colon | UC |
| 7 | 41 | Male | Colon | UC |
| 8 | 68 | Male | Colon | UC |
| 9 | 48 | Male | Colon | UC |
| 10 | 59 | Male | Colon | UC |

**Table S2 Antibodies for immunoblotting**

| Antibody | Cat. | Company | Dilution |
| --- | --- | --- | --- |
| Phospho-Stat3 (Tyr705) | #9145 | Cell Signaling Technology | 1:1000 |
| Phospho-NF-κB p65 (Ser536) | #8242 | Cell Signaling Technology | 1:1000 |
| Actin | #3700 | Cell Signaling Technology | 1:1000 |
| Stat3 | #9139 | Cell Signaling Technology | 1:1000 |
| P65 | #4764 | Cell Signaling Technology | 1:1000 |
| Bax | sc-7480 | Santa Cruz | 1:800 |
| Bak | sc-1035 | Santa Cruz | 1:800 |
| Mcl-1 | sc-74436 | Santa Cruz | 1:800 |
| Bcl-xl | sc-8392 | Santa Cruz | 1:800 |
| Bcl-2 | sc-7382 | Santa Cruz | 1:800 |
| γ-H2AX | #7631 | Cell Signaling Technology | 1:1000 |
| Phosph-p53 | #9284 | Cell Signaling Technology | 1:1000 |
| p53 | #2524 | Cell Signaling Technology | 1:1000 |
| GOLM1 (human) | 15089-1-AP | Proteintech | 1:800 |
| GOLM1 (mouse) | sc-48101 | Santa Cruz | 1:800 |
| NICD | #3608 | Santa Cruz | 1:800 |
| N2ICD | #5372 | Santa Cruz | 1:800 |
| Lamin A/C | #4777 | Cell Signaling Technology | 1:1000 |
| EEA | #48453 | Cell Signaling Technology | 1:1000 |
| HRP Goat Anti-Rabbit IgG (H+L) | AS014 | Abclonal | 1:4000 |
| HRP Rabbit Anti-Goat IgG (H+L) | AS029 | Abclonal | 1:4000 |
| HRP Goat Anti-Mouse IgG (H+L | AS003 | Abclonal | 1:4000 |

**Table S3 Primers sequences for Real Time quantitative PCR**

| *mIL-6* | F: TACCACTTCACAAGTCGGAGGC, R: CTGCAAGTGCATCATCGTTGTTC; |
| --- | --- |
| *mIL-1β* | F: TGGACCTTCCAGGATGAGGACA, R: GTTCATCTCGGAGCCTGTAGTG; |
| *mTNF-α* | F：GGTGCCTATGTCTCAGCCTCTT, R: GCCATAGAACTGATGAGAGGGAG; |
| *mCCL2* | F: AGAATCACCAGCAGCAAGTGTCC, R: TCCTGAACCCACTTCTGCTTGG; |
| *mZO-1* | F: GTTGGTACGGTGCCCTGAAAGA, R: GCTGACAGGTAGGACAGACGAT; |
| *mClaudin-1* | F: GGACTGTGGATGTCCTGCGTTT, R: GCCAATTACCATCAAGGCTCGG |
| *mJAMA1* | F: CACCTACTCTGGCTTCTCCTCT, R: TGCCACTGGATGAGAAGGTGAC; |
| *mHes1* | F: GGAAATGACTGTGAAGCACCTCC, R: GAAGCGGGTCACCTCGTTCATG; |
| *mHes5* | F: CCGTCAGCTACCTGAAACACAG, R: GGTCAGGAACTGTACCGCCTC; |
| *mAtoh1* | F: CTGGTAAGGAGAAGCGGCTGTG, R: CCATTCACCTGTTTGCTGGAAGG; |
| *mSpdef* | F: CACGTTGGATGAGCACTCGCTA, R: AGCCACTTCTGCACGTTACCAG; |
| mKlf4 | F: CTATGCAGGCTGTGGCAAAACC, R: TTGCGGTAGTGCCTGGTCAGTT; |
| mMuc2 | F: GCCCGTGGAGTCGTACGTGC, R: TTGGGGCAGAGTGAGGCGGT; |
| mChga | F: AAGTGCGTCCTGGAAGTCATCTC, R: GCTTGGCTTTTCTGGCTTGC; |
| mActin | F: CATTGCTGACAGGATGCAGAAGG, R: TGCTGGAAGGTGGACAGTGAGG; |
| hGolm1 | F: TGGCCTGCATCATCGTCTTG, R: CCCTGGAACTCGTTCTTCTTCA; |
| hHes1 | F: GGAAATGACAGTGAAGCACCTCC, R: GAAGCGGGTCACCTCGTTCATG; |
| hHes5 | F: TCCTGGAGATGGCTGTCAGCTA, R: CGTGGAGCGTCAGGAACTGCA; |
| hAtoh1 | F: CCTTCCAGCAAACAGGTGAATGG, R: GAACGACGGGATAACATTGCGC; |
| hCCND1 | F: GCTGCGAAGTGGAAACCATC, R: CCTCCTTCTGCACACATTTGAA; |
| hMyc | F: CCTGGTGCTCCATGAGGAGAC, R: CAGACTCTGACCTTTTGCCAGG; |
| hSmad1 | F: AGAGACTTCTTGGGTGGAAACA, R: ATGGTGACACAGTTACTCGGT; |
| hSmad4 | F: CTACCAGCACTGCCAACTTTCC, R: CCTGATGCTATCTGCAACAGTCC; |
| hGli1 | F: AGCGTGAGCCTGAATCTGTG, R: CAGCATGTACTGGGCTTTGAA; |
| hGli2 | F: CTGCCTCCGAGAAGCAAGAAG, R: GCATGGAATGGTGGCAAGAG ; |
| hActin | F: CACCATTGGCAATGAGCGGTTC, R: AGGTCTTTGCGGATGTCCACGT. |
